# Genomic targets of the IRE1-XBP1s pathway in mediating metabolic adaptation in epithelial plasticity

**DOI:** 10.1093/nar/gkad077

**Published:** 2023-02-11

**Authors:** Dianhua Qiao, Melissa Skibba, Xiaofang Xu, Allan R Brasier

**Affiliations:** Department of Medicine, University of Wisconsin-Madison School of Medicine and Public Health (SMPH), Madison, WI 53705, USA; Department of Medicine, University of Wisconsin-Madison School of Medicine and Public Health (SMPH), Madison, WI 53705, USA; Department of Medicine, University of Wisconsin-Madison School of Medicine and Public Health (SMPH), Madison, WI 53705, USA; Department of Medicine, University of Wisconsin-Madison School of Medicine and Public Health (SMPH), Madison, WI 53705, USA; Institute for Clinical and Translational Research (ICTR), University of Wisconsin-Madison, Madison, WI 1053705, USA

## Abstract

Epithelial mesenchymal plasticity (EMP) is a complex cellular reprogramming event that plays a major role in tissue homeostasis. Recently we observed the unfolded protein response (UPR) triggers EMP through the inositol-requiring protein 1 (IRE1α)–X-box-binding protein 1 spliced (XBP1s) axis, enhancing glucose shunting to protein N glycosylation. To better understand the genomic targets of XBP1s, we identified its genomic targets using Cleavage Under Targets and Release Using Nuclease (CUT&RUN) of a FLAG-epitope tagged XBP1s in RSV infection. CUT&RUN identified 7086 binding sites in chromatin that were enriched in AP-1 motifs and GC-sequences. Of these binding sites, XBP1s peaks mapped to 4827 genes controlling Rho-GTPase signaling, N-linked glycosylation and ER-Golgi transport. Strikingly, XBP1s peaks were within 1 kb of transcription start sites of 2119 promoters. In addition to binding core mesenchymal transcription factors SNAI1 and ZEB1, we observed that hexosamine biosynthetic pathway (HBP) enzymes were induced and contained proximal XBP1s peaks. We demonstrate that IRE1α -XBP1s signaling is necessary and sufficient to activate core enzymes by recruiting elongation-competent phospho-Ser2 CTD modified RNA Pol II. We conclude that the IRE1α-XBP1s pathway coordinately regulates mesenchymal transcription factors and hexosamine biosynthesis in EMP by a mechanism involving recruitment of activated pSer2-Pol II to GC-rich promoters

## INTRODUCTION

Mucosal surfaces are constantly exposed to environmental toxicants, injury and infectious organisms, triggering epithelial mesenchymal plasticity (EMP) to promote wound healing and repair (1). EMP is a coordinated cell-state change driven by two competing intercellular signaling pathways- one, promoting epithelial mesenchymal transition (EMT), and the second reversing the process through MET (2). As a result of mesenchymal transition, differentiated epithelial surfaces express mesenchymal contractile proteins, disrupt adherens junctions and lose apical-basal polarity to promote cellular migration, proliferation and mucosal repair. Although important in acute tissue injury, chronic activation of EMP results in fibrosing organ dysfunction. A more detailed understanding of the complex intracellular signaling pathways controlling EMP is needed.

A body of work has shown that both RNA viral replication and epithelial growth factors trigger EMP through a common pathway converging on the master regulatory pathway of innate immunity, IκB kinase (IKK)-NFκB (3–6). IKK is a signal integrator of activated TGFβ and liganded pattern recognition receptors that mediates a cascade of phospho-signaling networks (5). Downstream, activated NFκB complexes with a histone acetyl transferase, Bromodomain containing protein 4 (BRD4), translocating into the nucleus where it functions as an epigenetic regulator to activate mesenchymal regulatory factors through a process of transcriptional elongation (4,7). These EMT regulators include the Snail family repressor 1 (SNAI1), Zinc Finger E-Box Binding Homeobox 1 (ZEB1) and Wnt (3). As epithelial cells transition from partial into stable EMT, enhanced expression of SNAI1 disrupts the SNAI1-ZEB1 miRNA autoregulatory feedback loop (8), resulting in expression of additional downstream cliques of mesenchyme-specifying transcription factors (7). Consequently, robust expression of ECM components fibronectin (FN), MMP and the epithelial growth factors, TGFβ and IL6 occurs (9). Because differentiated epithelial cells are non-secretory cells, this abrupt production of ECM components is a potent inducer of cellular apoptosis through endoplasmic reticulum (ER) stress. How the epithelial cell adapts to ER stress in the EMP transition is not fully understood.

Using infections with the orthopneumovirus, respiratory syncytial virus (RSV), we recently discovered an adaptive metabolic pathway initiated by unfolded protein response (UPR) that results in protein N glycosylation (10,11). Here, the inositol-requiring protein 1 (IRE1α)–X-box-binding protein 1 (XBP1) arm of the UPR is selectively activated (11) by the rapid influx of RSV-encoded glycoproteins that dissociates the ER-resident immunoglobulin binding chaperone (BiP/Grp78) from the IRE1α endonuclease (12), triggering autophosphorylation and activation. IRE1α alternatively splices a 26 nt fragment from XBP1u mRNA, whose translation forms the bZIP-class XBP1s transcription factor that translocates into the nucleus to interact with chromatin-bound transcriptional regulators. In addition to activating the SNAI1 and ZEB1 mesenchymal transcription factors, IRE1-XBP1s signaling shifts glucose flux from glycolysis to uridine diphosphate N-acetylglucosamine (UDP-GlcNAc) production (11,13). UDP-GlcNAc is a rate limiting substrate for protein N-glycosylation; its increased production during the UPR enables native folding and secretion of basement membrane modifying enzymes involved in collagen crosslinking, elastin degradation and assembling a fibronectin-enriched matrix, remodeling the basement membrane (13). The mechanistic details how IRE1α-XBP1s pathway activates N glycosylation is not fully understood.

Gene regulatory networks controlled by the IRE1α-XBP1s pathway are under cell type-and stimulus-selective modulation (14,15). To understand the role of this pathway in epithelial EMP, we sought to systematically identify the gene regulatory network activated by XBP1s using Cleavage Under Targets and Release Using Nuclease (CUT&RUN) in RSV infected cells. Of the 7086 high confidence XBP1s binding sites, 2119 map to proximal promoters within 1 kb of the transcription start site. Genes containing inducible XBP1s binding and who were functionally regulated by RSV included sequential enzymes constituting the core HBP pathway, including Glutamine-Fructose-6-Phosphate Transaminase 1 and 2 (GFPT1/2); Glucosamine-Phosphate N-Acetyltransferase 1 (GNPNAT1), Phosphoglucomutase 3 (PGM3) and UDP-N-Acetylglucosamine Pyrophosphorylase 1 (UAP1). These data indicate that the IRE1α-XBP1s is a direct gene regulator of the core HBP pathway by recruiting activated RNA polymerase II to core enzymes in hexosamine biosynthesis.

## MATERIALS AND METHODS

### Human small airway epithelial cell (hSAEC) culture and treatment

Immortalized primary hSAECs were obtained from American Type Culture Collection (ATCC, Gaithersburg, MD, USA) and grown in SAGM small airway epithelial cell growth medium (Lonza, Walkersville, MD, USA) in 5% CO_2_ (11,16). Sucrose cushion purified RSV Long strain was prepared and titered using methylcellulose plaque assay (11). hSAECs were infected at a multiplicity of infection (MOI) of 1.0 for 24 h prior to harvest. For induction of the UPR, hSAECs were treated for indicated times with various standardly used doses of 0.5–0.5 μg/ml tunicamycin (TM) or 50 nM thapsigargin (Tg). The selective IRE1α RNAse inhibitor KIRA8 (MedChemExpress, South Brunswick Township, NJ, USA) was added directly to the culture medium at a concentration of 10 μM where indicated (17).

### RNA isolation and quantitative RT-PCR (Q-RT-PCR)

Total cellular RNA was isolated using RNeasy kit with on-column DNase digestion (Qiagen). Synthesis of complementary DNAs (cDNAs) was done with SuperScript III First Strand cDNA Synthesis Kit (Thermo Scientific). Q-RT-PCR assays were performed using iTaq SYBR Green Master Mix (Bio-Rad) and gene-specific primers (Table 1). Data are presented as fold change using the △△Ct method normalizing to *PPIA* as internal control.

**Table 1.** Quantitative RT-PCR primers

Gene	Forward	Reverse
GFPT1	5′-CTGAGATTGGTGTGGCCAGT-3	5′-GGCAGCCGTTTCAATCCAAG-3′
GFPT2	5′-GGGCATCCTGAGCGTGATTC-3′	5′-CCATGTAGCATCCCTGCTGT-3′
GNPNAT1	5′-ATCCTGGAGAAGGCTTGGTT-3′	5′-CAGAGTTGCCGTAGCAACAA-3′
PGM3	5′-TCATGTTTCGCATGGGATTA-3′	5′-AAACAGGTGGCATGTTCCTC-3′
UAP1	5′-GAGGCATTTGGAGCATTCAT-3′	5′-TCCGTCTGAGCTTCGTTTTT-3′
XBP1s	5′-GCTGAGTCCGCAGCAGG-3′	5′-CTCTGGGGAAGGGCATTTGA-3′
XBP1u	5′-ACTCAGACTACGTGCACCTCT-3′	5′-CTGGGTCCAAGTTGTCCAGAA-3′
XBP1us (Total)	5′-GTCACCCCTCCAGAACATCTC-3′	5′-TCTGGGGAAGGGCATTTGAA-3′
IRF1	5′-GAGGAGGTGAAAGACCAGAGCA-3′	5′-TAGCATCTCGGCTGGACTTCGA-3′
CCL20	5′-AAGTTGTCTGTGTGCGCAAATCC-3′	5′-CCATTCCAGAAAAGCCACAGTTTT-3′
CXCL1	5′-AGCTTGCCTCAATCCTGCATCC-3′	5′-TCCTTCAGGAACAGCCACCAGT-3′
IRE1	5′-TGTGTCAACGCTGGATGGAA-3′	5′-TCCACATGTGTTGGGACCTG-3′
PPIA	5′-CGCGTCTCCTTTGAGCTGTT-3′	5′-CCATAGATGGACTTGCCACCA-3′
RSV N	5′-AAGGGATTTTTGCAGGATTGTTT-3′	5′-TCCCCACCGTAACATCACTTG-3′

### Short hairpin RNA (shRNA) gene silencing

shRNA silencing was performed using lentivirus transduction. For silencing *XBP1* or *IRE1*, five Sigma Mission shRNA lentiviral vectors were generated and populations of transduced hSAECs were selected in 2 μg/ml puromycin. Gene knock-down efficiencies of the shRNAs were assessed by Q-RT-PCR in the absence or presence of RSV. The most effective silencing lentivirus was then selected for subsequent experiments. The target sequences of the shRNAs used were: *XBP1*, 5′-GCCTGTCTGTACTTCATTCAA-3′; *IRE1*, 5′-GCAGGACATCTG GTATGTTAT-3′. A non-targeting luciferase Sigma Mission shRNA lentiviral vector (Sigma, cat. SHC007) was used as negative control.

### FLAG-XBP1s expression

The FLAG-XBP1s expression vector was constructed by cloning 3xFLAG peptide-tagged human XBP1s cDNA fragment into a lentiviral vector driven by a CMV promoter. The 3xFLAG tag coding sequence (GAC TAC AAA GAC CAT GAC GGT GAT TAT AAA GAT CAT GAC ATC GAT TAC AAG GAT GAC GAT GAC AAG) was inserted by PCR into human XBP1s cDNA immediately after the start codon of XBP1s. Lentiviruses expressing FLAG-XBP1s (FXBP1s) and the empty vector (pCT) were generated by standard lentivirus preparation method using calcium phosphate precipitation transfection. Specifically, following overnight transfection of HEK293T cells in Dulbecco's Modified Eagle Medium (DMEM) containing 5% fetal bovine serum (FBS), the cells were cultured for 48 h in SAGM and the virus-containing medium was collected and stored at -80°C after clearance of cell and cell debris. To transduce hSAECs, the lentivirus-containing SAGM were incubated with sub-confluent hSAECs overnight in the presence of 10 μg/ml polybrene at an MOI of 2.0. The cells were incubated in fresh SAGM for additional 24 h prior to further treatment.

### Reporter assay

Sub-confluent SAECs in 48-well plate were co-transfected by UPR luciferase reporter and FLAG-XBP1s expression vector using Lipofectamine 3000 (Invitrogen) according to manufacturer's instruction. Specifically, 100 ng of specific UPR luciferase reporter, 10 ng of the minimal promoter-driven NanoLuc luciferase reporter, pNL3.1 (Promega) as the internal control, and increasing amounts (0 to 300 ng) of FLAG-XBP1s expression vector were used. The difference in total DNA amount was compensated by empty cDNA vector. The UPR luciferase reporters used include pGL4-UPRE-luc2P-Hygro, pGL4-ERSE1-luc2P-Hygro and pGL4-ERSE2-luc2P-Hygro, minimal promoters driven by three copies of previously identified UPR cis-elements, unfolded protein response element (UPRE) and ER stress response element (ERSE)-I and -II (Addgene plasmid numbers 101788, 101789 and 101790, respectively) (18). The transfected cells were cultured for 48 h prior to harvest in 100 μl of Passive Lysis Buffer (Promega, Fitchberg, WI, USA). Dual luciferase assay was carried out using Nano-Glo Dual Luciferase Assay System (Promega) per manufacturer's instruction. 40 μl of cell lysate was used in each reaction. The firefly luciferase activities produced by the UPR luciferase reporters were normalized by corresponding NanoLuc luciferase activities produced by pNL3.1 and presented as mean ± 25-75% range.

### Western blot

Cells were trypsinized, pelleted and washed with cold phosphate-buffered saline (PBS). Nuclei were isolated as described in CUT&RUN below, lysed in cell lysis buffer (10 mM Tris, pH 7.5, 100 mM NaCl, 1 mM EDTA, 1 mM EGTA, 20 mM Na_4_P_2_O_7_, 1 mM β-glycerol phosphate, 0.1% SDS, 0.5% sodium deoxycholate, 1% Triton X-100, 10% glycerol, 2 mM activated Na_3_VO_4_, 1 mM NaF and fresh added 1× protease inhibitor cocktail), and centrifuged at 16 000 × g at 4°C for 10 min to remove nuclear debris. Proteins were resolved on 4–15% Criterion TGX precast SDS-PAGE gels (Bio-Rad) and transferred to PVDF membranes with Bio-Rad Trans-Blot Turbo transfer system. Primary antibodies used were anti-FLAG M2 (Sigma Aldrich F1804), anti-XBP1s (Clone 143F, BioLegend) and anti-TBP (TATA-box binding protein) as a loading control. Blots were imaged and quantified using ImageJ.

### Immunofluorescence microscopy

hSAECs were plated on coverslips, and infected or treated as indicated. Afterwards, cells were fixed with 4% paraformaldehyde, permeabilized with 0.2% Triton X-100, blocked with 10% goat serum and incubated with primary antibody overnight. Primary antibodies used were anti-FLAG M2. On the second day, coverslips were washed and incubated with Alexa fluor goat secondary antibody. After 1 h, cells were washed and mounted using ProLong Diamond Antifade Mountant with 4′,6-diamidino-2-phenylindole (DAPI, Thermo Fisher). The cells were visualized in an ECHO fluorescence microscope.

For tissue staining, formaldehyde-fixed paraffin-embedded (FFPE) mouse lung sections were deparaffinized, rehydrated, heated in a steamer in Tris-EDTA buffer (pH 9.0) for antigen retrieval and blocked overnight at 4°C in 10% goat serum in PBST (PBS with 0.05% Tween 20), followed by overnight incubation at 4°C with anti-XBP1s ribbit polyclonal antibody (24868-1-AP, Proteintech, IL) diluted at 1:100 in the blocking buffer. Slides were washed in PBST (5 times, 5 min each), and incubated with Alexa Fluor 647-conjugated goat-anti-rabbit 2nd antibody (Thermo) diluted at 1:1000 in the blocking buffer for 1 h at room temperature. After washing 5 × 5 min in PBST, the sections were mounted using ProLong Diamond Antifade Mountant with DAPI (Thermo) and imaged.

### Two-step chromatin IP (XChIP)-quantitative genomic PCR (Q-gPCR)

Protein–protein cross-linking was performed with DSG (2 mM, 45 min at 22°C) followed by protein–DNA cross-linking with formaldehyde (19). Equal amounts of sheared chromatin were immunoprecipitated (IPed) overnight at 4°C. IPs were collected with 40 μl protein-G magnetic beads (Dynal Inc), washed and eluted in 250 μl elution buffer for 15 min at 65ºC. Following de-crosslinking and phenol-chloroform DNA precipitation, gene enrichment was determined by Q-gPCR using region-specific PCR primers (Table 2). SNAI1 primers used were previously reported (11). The fold change of DNA in each IP was determined by normalizing the absolute amount to input DNA reference and calculating the fold change relative to that amount in unstimulated cells (19).

**Table 2.** Q-gPCR primers for XChIP assay

Genic region	Forward	Reverse
GFPT2 enhancer	5′-GGAGTTGGGACGGAAAGTCA-3′	5′-GAAGCTCACCCTTGCCACTA-3′
GNPNAT1 promoter	5′-GGGGTAGGAGCCTAGGAAAA-3′	5′-GCGTGGGAAATGAGACAGTT-3′
PGM3 promoter	5′-GCCTAGGTCCACGTACCAGA-3′	5′-CTCGGAGTTGAGAAGGGAGA-3′
UAP1 promoter	5′-AGTGGGACAGGAGATCGTTG-3′	5′-AGAGAGGGGAAACCCAGAAA-3′
GFPT1 promoter	5′-CCTCCTCTACCCCTCCACAA-3′	5′-CCCAAAGGTCAGCCTCTCTG-3′

### Cleavage under targets and release using nuclease (CUT&RUN)

hSAECs transduced by FLAG-XBP1s (FXBP1s) lentivirus (MOI = 2.0, 48 h) were treated in triplicate replicates with or without RSV infection (MOI = 1.0, 24 h). Untransduced mock cells were negative control. 4 × 10^6^ trypsinized cells were aliquoted and incubated on ice for 10 min in 1 ml of nuclear extraction buffer (20 mM HEPES, pH 7.9, 10 mM KCl, 0.1% Triton X-100, 20% glycerol, 1× cOmplete proteinase inhibitor, 1× protein phosphatase inhibitor cocktail and 0.5 mM spermidine), the released nuclei were pelleted at 600 × g, 5 min at 4°C. A basic wash buffer (WB) consisting of 20 mM HEPES, pH7.5, 150 mM NaCl, 0.05% Triton X-100, 0.1% BSA, 1× cOmplete proteinase inhibitor, 1× protein phosphatase inhibitor cocktail and 0.5 mM spermidine was used throughout. Between incubation steps, the nuclei were pelleted at 600 × g, 3 min at 4°C. Prior to antibody binding, the isolated nuclei were incubated with nutation at 4°C for 5 min in WB containing 2 mM EDTA, followed by nutation at 4°C for 25 min in WB. The nuclei were resuspended in antibody buffer produced by diluting 5 μg of anti-FLAG M2 antibody (Sigma) in 500 μl of Triton X-100-free WB, and incubated with nutation overnight at 4°C. After washing three times in 500 μl of WB on ice (10 min each time), the nuclei were incubated at 4°C for 1 h in 50 μl of WB containing 2.5 μl of EpiCypher pAG-MNase 20× stock (EpiCypher, NC). Washing as above, targeted chromatin cleavage was then conducted by incubating the nuclei on ice for 1 h in 150 μl of BSA-free WB containing 2 mM CaCl_2_. The cleavage was terminated by adding 150 μl of stop buffer (300 mM NaCl, 20 mM EDTA, 4 mM EGTA and 0.5 ng of *E. coli* Spike-in DNA (EpiCypher, NC) per 150 μl). The samples were further incubated with nutation at 4°C for 1 h, centrifuged at 16 000 × g for 5 min at 4°C and the supernatant was collected. Phenol-chloroform DNA precipitation with 80 μg of glycogen was performed and the Cut&Run-enriched DNA was dissolved in 20 μl of 0.1× TE buffer. Following DNA quantitation by a Qubit fluorometer, CUT&RUN DNA libraries were prepared using NEBNext Ultra II DNA Library Prep Kit for Illumina (NEB, MA) per the manufacturer's instruction, with modification in SPRI bead clearance of adaptor ligation and library amplification reactions to retain small-sized DNA fragments. The quality of the resultant DNA libraries was confirmed by Agilent TapeStation HS DNA assay (Agilent, Santa Clara, CA, USA), and paired-end Illumina NGS was carried out on NovaSeq 6000 with 5 million reads per sample.

Fastqc files were analyzed for quality metrics (Phred Score, GC content, duplication) using fastQC. Adapters were trimmed using TrimGalore. Genome alignment was by MACS2 to the GRCh38.p13 (hg38) genome assembly (NCBI) (20). Peak calling was using SEACR with top 20% peaks cut-off (21). Differential peak occupancy was determined using DIFFBIND v 4.2 using DESEQ2 (22). Differential enrichment was using adjusted *P* values (*p*_Adj_) at <0.05 to control for multiple hypothesis testing. Superenhancer analysis was using ROSE (23,24). Enrichment analysis of known transcription factors was performed using position-weight matrices using hg38 as background with HOMER software v 4.11 (25).

### Animal model of RSV infection

Animal experiments were performed in accordance with the NIH Guide for Care and Use of Experimental Animals and approved by the University of Wisconsin at Madison Institutional Animal Care and Use Committee (approval no. M006067). Wild-type 7-week old C57BL/J6 black mice (both genders) were administered RSV (RSV long strain; 10^7^ PFU) or vehicle (PBS) via the intranasal route and euthanized at peak of RSV replication on day 5. Lung tissues were collected for preparing FFPE tissue slides for immunofluorescence staining and for Q-RT-PCR analysis of total lung RNA.

### Statistical analyses

Statistical analyses were performed with Graph Pad Prism 9 (GraphPad Software, San Diego, CA). Results are expressed as mean ± SD. Normality and equal variance tests were performed to determine appropriate application of parametric statistical analyses. For multiple group experiments, ANOVA was used with post-hoc Tukey T-tests for group-wise comparison between treatments. *P* values <0.05 were considered to be statistically significant.

## RESULTS

### XBP1s is activated by RSV infection in vivo

**Figure 1.**
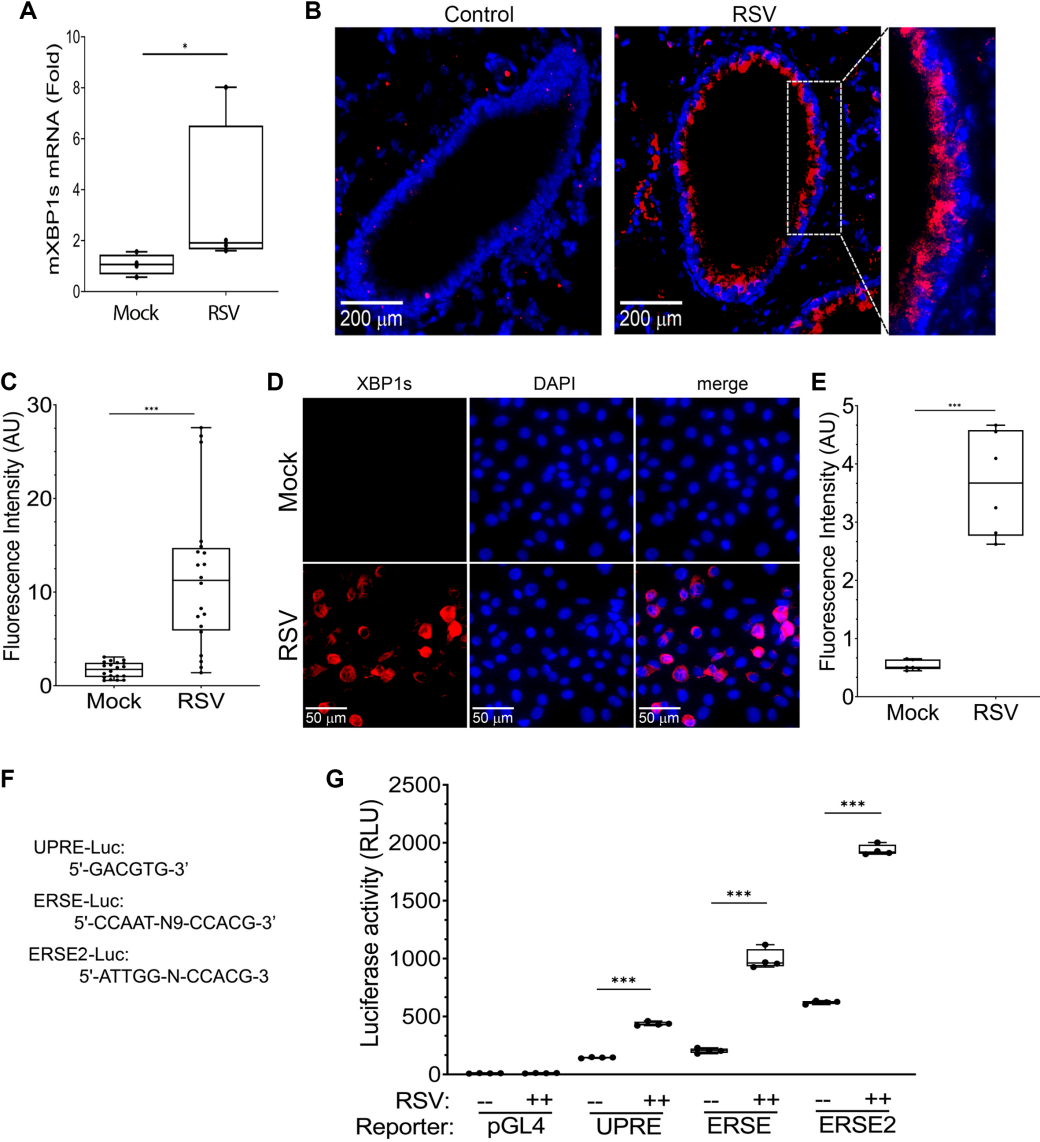
RSV activates XBP1 splicing. (**A**) RSV induces formation of XBP1s mRNA. C57BL/6J black mice were infected with RSV for 5 days. Lung total RNA was harvested and used to measure XBP1s by Q-RT-PCR. Shown is fold change of mXBP1s mRNA normalized to mGAPDH mRNA as internal control. Symbols are individual mice; the box is 25%–75% interquartile ranges, with median ± ranges. *P < 0.05, Mann-Whitney test. (**B**) RSV induces XBP1s in lung epithelial cells. Mouse lungs from experiment in 1A were formalin fixed and subjected to immunofluorescence using anti-XBP1s Ab (Alexa Fluor 647, red). Nuclei were counterstained with 4′,6-diamidino-2-phenylindole (DAPI, blue). Inset, enlarged section of small bronchiole. Scale bar of 200 microns is shown. (**C**) Quantitation of XBP1s fluorescence. Fluorescence intensity of high power fields was quantified in FIJI. Symbols are individual data points from separate fields from four independent sections. ***P < 0.001, t test. (**D**) RSV induces XBP1s formation in hSAECs. hSAECs were uninfected (mock) or RSV infected (MOI = 1, 24 h), fixed and stained for immunofluorescence with anti-human XBP1s (red). Nuclei were counter stained with DAPI (blue). Scale bar of 50 microns is shown. (**E**) Quantitation of XBP1s fluorescence intensity. Fluorescence intensity of random fields was quantified in FIJI. ***P < 0.001, *t* test. (**F**) Unfolded protein response (UPR) target sequences. Shown are core sequences used in UPR-driven reporters. UPRE, UPR element; ERSE, ER stress response element. (**G**) RSV activates UPR element-driven transcription. hSAECs were co-transfected with luciferase reporter plasmids containing three copies of UPRE, ERSE and ERSE2, respectively, and pNL3.1 (Promega) as the internal control. Shown is normalized firefly luciferase activities to internal control NanoLuc luciferase activities. Each symbol is an independent replicate. ***P < 0.001, post-hoc analysis.

The IKK signalsome integrates diverse signals from pattern recognition receptors and epithelial growth factors to activate EMP (3,5,26). To identify a system for understanding how pattern recognition receptors activate EMP and the homeostatic XBP1s pathway, we investigated the effect of RSV infection on XBP1s expression. Previous work *in vitro* has shown that influx of RSV F and G glycoproteins into the ER displaces the immunoglobulin binding chaperone (BiP/Grp78) from IRE1 (12), triggering its autophosphorylation and activation. To establish this effect *in vivo*, we examined whether XBP1s formation occurred *in vivo*. Wild type C57BL/J6 mice were infected with RSV via the intranasal route which produces a time-dependent activation of NFκB activation, innate responses and epithelial plasticity (27). Relative to mock-infected mice, we observed that RSV infection induced a 1.9-fold activation of XBP1s splicing in total lung RNA (*P* < 0.03, Mann–Whitney test, Figure 1A). To confirm that the protein was expressed, lung tissues were fixed and stained with anti-XBP1s antibodies. We noted a dramatic induction of XBP1s in the cytoplasm and nuclei of lining epithelial cells (Figure 1B). When quantitated, XBP1s staining increased from a baseline of 1.7 ± 0.8 arbitrary fluorescence units (AU) to 11.6 ± 7.9 AU (*n* = 20 fields, *P* < 0.0001, Figure 1C). These data demonstrate that the UPR is activated in small airway epithelial cells during the acute phase of RSV infection.

We next examined the response of human small airway epithelial cells (hSAECs) to RSV. Earlier we identified the activation of IRE1α -XBP1s signaling *in vitro* in a well-established model of human small airway epithelial cells (hSAECs) that induce innate signaling, EMP and metabolic reprogramming pathways in response to paramyxovirus infection (3,6,16,28,29). RSV infection produced a robust increase in perinuclear and nuclear XBP1s staining from 0.54 ± 0.8 to 3.7 ± 0.9 AU (*P* < 0.0001, *n* = 6 fields, Figure 1D, E).

To demonstrate whether this change in XBP1s was functionally active, we asked whether RSV activated a UPR-driven reporter. The basic leucine zipper DNA binding motif of XBP1s binds to heterogenous sequences, three distinctive XBP1 binding DNA elements were previously identified in ER-associated XBP1s target genes, they include unfolded protein response element (UPRE), GACGTG (30), and the ER stress response element (ERSE), CCAAT(N_9_)CCACG, or ERSE2, ATTGG(N)CCACG (31). Empty control (pGL4), UPRE-Luc, ERSE-Luc or ERSE2-Luc reporters were transfected into hSAECs, and mock or RSV-infected (MOI = 1, 24 h). Relative to the negligible Luciferase activity in empty pGL4 vector, RSV increased UPRE activity by 3-fold from 145 ± 5 to 438 ± 17 normalized relative light units (RLUs; *P* < 0.001, Figure 1G). Similarly ERSE-driven reporter was increased 4.9-fold from 204 ± 20 to 992 ± 87 RLUs, and ERSE2-driven reporter activity was increased 3.1-fold from 622 ± 14 to 1,936 ± 45 RLUs (Figure 1G). These data establish that XBP1s pathway was functionally activated in RSV-infected hSAECs as a biologically relevant model.

### Transcriptionally competent XBP1s expression

**Figure 2.**
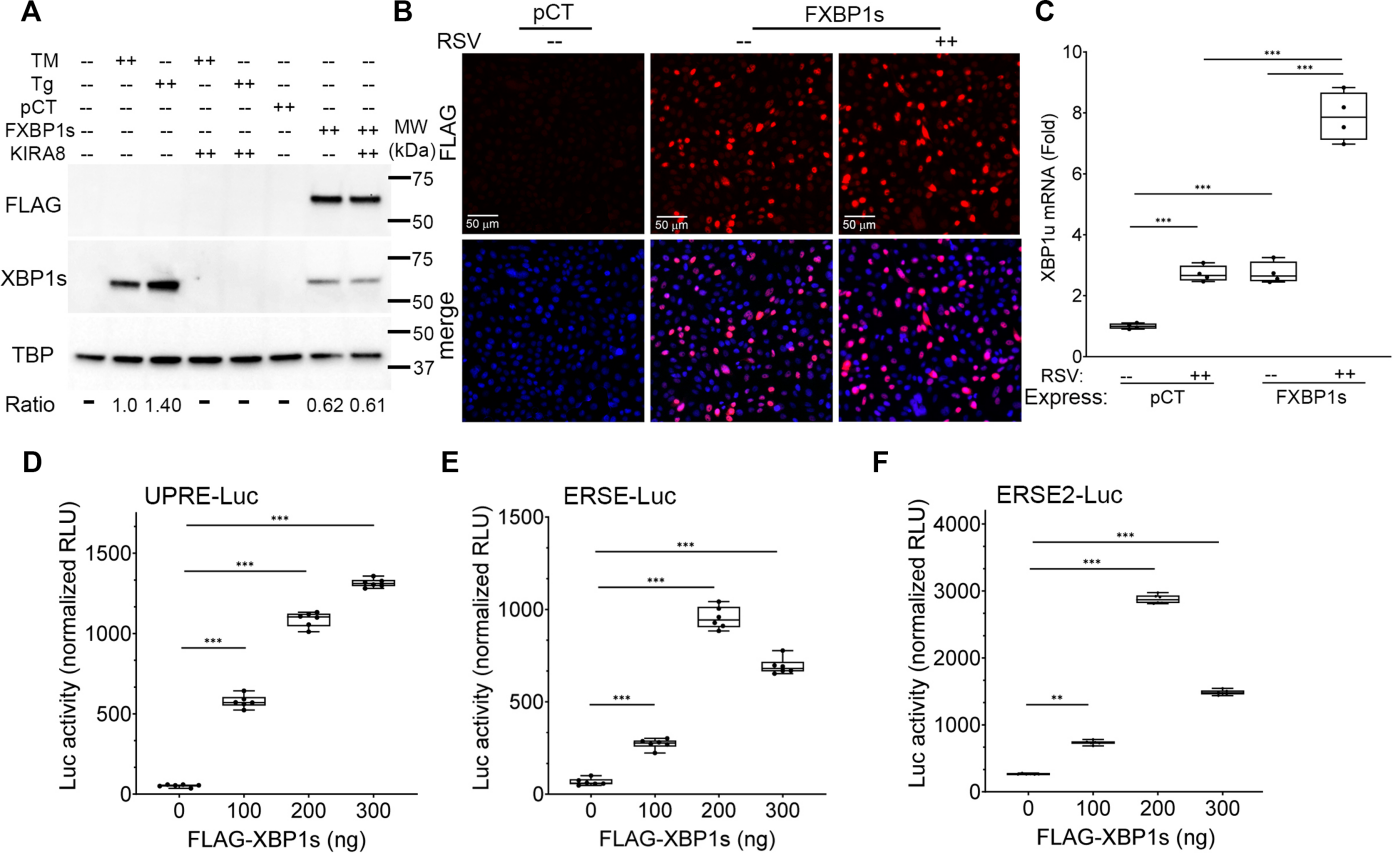
Expression of functionally active FLAG epitope-tagged XBP1s (FXBP1s). (**A**) FXBP1s expression. Western immunoblot of hSAECs transduced with empty (pCT) or FXBP1s-expressing lentiviral vector at an MOI of 2.0 for 48 h, or mock-treated or treated with tunicamycin (TM, 0.5 μg/ml for 8 h) or thapsigargin (Tg, 50 nM for 6 h) in the absence or presence of KIRA8 (10 μM). Nuclear extracts were prepared and stained with anti-FLAG M2 or anti-XBP1s antibodies. TBP was used as loading control. MW, molecular weight markers (kDa) are shown. Ratio, the relative intensities of the immunoblotting signals quantitated by FIJI with normalization to TBP. Note the intense anti-FLAG M2 staining of ∼60 kDa protein encoded by the FXBP1s expression vector and that TM and Tg have induced significantly higher levels of XBP1s than that produced by FXBP1s transduction. (**B**) Immunofluorescence microscopy was performed to assess transduction efficiency and nuclear translation. Cells were fixed and stained with anti-FLAG M2 (red) and counter-stained with DAPI (blue). Note the >50% transduction of the cell population and nuclear localization. Scale bar of 50 μm is shown. (**C**) Activation of endogenous XBP1s target genes. XBP1 is auto-regulated by XBP1s, where XBP1s binds to its own promoter initiating mRNA expression (11). Shown is XBP1u mRNA expression in pCT or FXBP1s-transduced cells. Shown is fold change of XBP1u mRNA normalized to PPIA mRNA as internal control. Symbols are individual replicates; horizontal line, mean; the box is 25–75% interquartile ranges. ***P < 0.001, post-hoc analysis. (D–F) FXBP1s transactivates UPRE-driven reporters. (**D**) UPRE-, (**E**) ERSE- and (**F**) ERSE2-driven luciferase reporters were co-transfected with increasing amounts of FXBP1s expression vector (in ng). Shown is normalized firefly luciferase activities produced by the UPR reporters to internal control NanoLuc luciferase activities produced by pNL3.1 (Promega). Each symbol is an independent replicate. ***P < 0.001, post-hoc analysis.

In preliminary studies, we found that antibodies to XBP1s are of low affinity and specificity (cross-reactive with unspliced XBP1) precluding precise identification of XBP1s binding sites using CUT&RUN. To circumnavigate this problem, we selectively expressed a FLAG epitope-tagged XBP1s (FXBP1s) in a lentivirus expression vector, by which the expression levels of FXBP1s can be controlled by titration of the multiplicity of infection (MOI). In preliminary studies we observed that the epitope-tagged FXBP1s was detected in transduced cells as a ∼60 kDa nuclear protein that cross-reacted with anti-XBP1s antibody. To confirm that the FXBP1s expression an an MOI of 2 mimicked that of endogenous UPR activation, expression levels of FXBP1s were compared to XBP1s produced endogenously by UPR activation using standard treatments with tunicamycin (TM) or thapsigargin (Tg). In preliminary studies, dose-response and time course experiments were conducted to select optimal conditions for TM and Tg activation of the endogenous UPR. In hSAECs, peak UPR activation was observed with 0.5 μg/ml TM at 8 h, and 50 nM Tg at 6 h (Supplementary Figure S1), which were used for further Western blot analysis. Western blot was performed probing nuclear extracts with anti-FLAG or anti-XBP1s antibodies (Abs), using TATA Box Binding Protein (TBP) as a loading control. In untreated cells, XBP1s was not detectable (Figure 2A). By contrast, TM and Tg treatment induced robust accumulation of nuclear XBP1s. Here, we observed that Tg was a slightly stronger activator of XBP1s accumulation than TM (1.4-fold) consistent with the mRNA expression analysis (Supplementary Figure S1). The activation of XBP1s was completely inhibited by treatment with the IRE1α RNase selective inhibitor, KIRA8 (11,17). The FLAG staining was observed only in the FXBP1s-transduced cells (Figure 2A). We observed that the FXBP1s expression was ∼60% of the XBP1s level induced by TM and unaffected by the treatment with KIRA8 as expected (Figure 2A). Immunofluorescence microscopy was used to confirm that the FXBP1s protein was expressed in > 50% of the transduced hSAECs, where it largely concentrated in the nucleus, determined by co-localization with the nuclear DAPI marker (Figure 2B). These data indicate that FXBP1s expression was physiologically comparable to that produced by endogenous UPR activation by the IRE1α RNAse and localized to the nucleus.

To test whether this protein was able to functionally transactivate endogenous XBP1s-dependent target genes, we first analyzed the expression of endogenous unspliced XBP1 (XBP1u). XBP1u mRNA was selected as an XBP1s transactivation readout because our earlier work showed that *XBP1* gene is positively autoregulated by XBP1s binding to its own promoter during the UPR (11). We selectively quantitated XBP1u expression using an anchor PCR primer hybridizing to the 26 nt intron spliced out by IRE1 RNase. We observed that expression of XBP1u mRNA in FXBP1s-transduced cells increased to 2.75 ± 0.36-fold over that of cells transduced with empty pCT vector (*P* = 0.0009, *n* = 4, post-hoc Tukeys). Moreover, the FXBP1s-induced XBP1u mRNA expression was further potentiated to 7.9 ± 0.8-fold versus 2.72 ± 0.3-fold in empty vector-transduced cells in response to RSV infection (Figure 2C; *P* < 0.0001).

To further confirm that the FXBP1s functionally transactivated UPR elements, we tested the effect of FXBP1s expression on the panel of UPR-driven reporter plasmids containing differential UPR DNA cis-elements (Figure 1F). We observed a robust, dose-dependent 13-fold increase of UPRE-driven reporter activity from 53 ± 8.6 to 1314 ± 26 normalized relative light units (RLUs) upon FXBP1s transduction (*P* < 0.0001, Figure 2D). Similarly, FXBP1s transactivated ERSE and ERSE2-driven reporters by 14.4- and 11-fold over no transactivator, respectively (both *P* < 0.0001; Figure 2E, F). In the ERSE and ERSE2-driven reporters, saturation of FXBP1s transactivation activity at 200 ng is probably due to limited NFY or ATF6 heterodimers in the mock-infected cells. Collectively, we interpret these data to indicate that FXBP1s expression was a functionally active transcriptional activator, and with homogeneous cellular expression, suitable for CUT&RUN profiling.

### CUT&RUN analysis of XBP1s binding in the hSAEC genome

**Figure 3.**
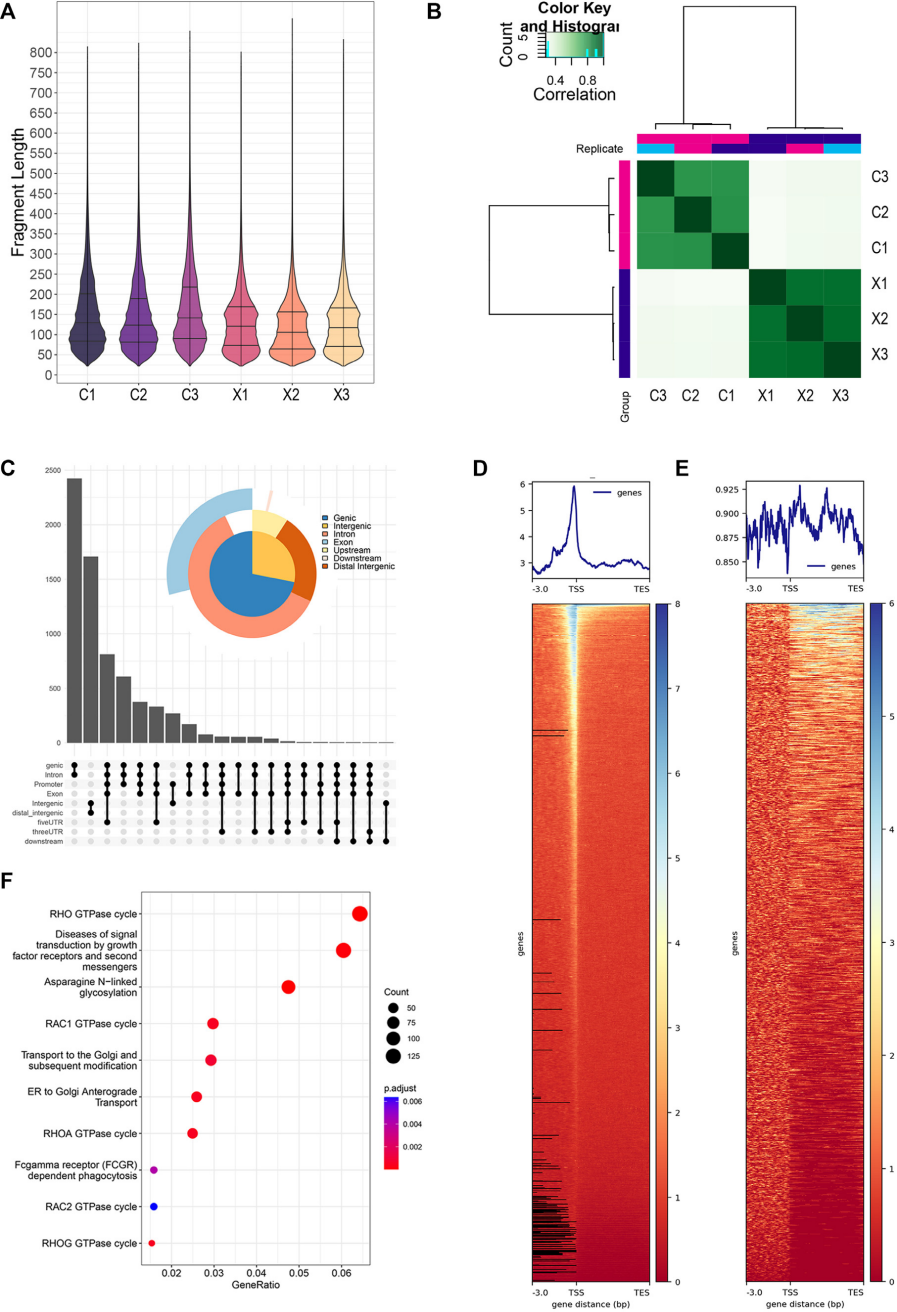
Distribution of FLAG-XBP1s binding in the hSAEC genome. (**A**) CUT&RUN analysis was applied to transduced cells FXBP1s versus empty vector, both treated with FLAG M2 Ab. Fragment length distribution of cleavage fragments is shown in violin plots after removal of adapters. Each replicate is shown. C, control; X, FXBP1s. Note the distinct 70 and 180 nt pattern in the FXBP1s fragments that are absent in the nonspecific cleavage pattern of the control. (**B**) Correlation plots of individual CUT&RUN sequencing. Note the high cross-correlation of the RSV-FXBP1s sequences with each other, distinct from that of control. (**C**) Annotation of FXBP1s binding sites to target genes. Shown is an upset plot of FXBP1s binding to genes. The upset plot provides information on correspondence of multiple peaks in a gene body. Note the marked enrichment of XBP1s binding to genic sequences. (**D**) XBP1s binding is enriched on proximal promoters. Heatmap of XBP1 binding to transcription start sites of annotated genes, normalized for gene length. Note the sharp peak enrichment relative to transcription start site (TSS). (**E**) XBP1s binding to genic sequences. Heatmap of XBP1s interaction with introns and gene bodies. (**F**) Gene pathways controlled by FXBP1s-bound genes within 3 kb of the transcription start site. Shown are genome ontologies ranked by the number of genes in a pathway (gene ratio) and by enrichment relative to genome (adjusted *P* value, padjust). In addition to cellular signaling, note the multiple entries for protein N glycosylation, ER processing and ER-Golgi transport. These pathways are shared with the genes bound by FXBP1 within 1 kb of the transcription start site (Supplementary Figure S2).

To maximize our ability to detect XBP1s binding sites, in addition to mock-infected FXBP1s expressing cells, CUT&RUN profiling was also performed in FXBP1s expressing cells infected with RSV, where the activation of IKK stabilizes the XBP1s protein (5). The NGS sequencing reads were subjected to quality, which indicated high-confidence base calling of >150 bp with Phred scores >30 (not shown). After trimming the sequencing primers, we analyzed the fragment size of the library. We observed that the FXBP1s-transduced cells produced fragment sizes binomially distributed at ∼70 and ∼160 nt in length. By contrast, control fragment sizes were monotonically distributed at ∼90 nt (Figure 3A). Examining sample similarity, we found that the DNA fragments co- clustered by treatment type, with control (Con) replicates and FXBP1s replicates (X) clustering together (Figure 3B). Statistically significant peaks occupied by FXBP1s were identified using DESEQ2 in DiffBind where 7086 occupied peaks in FXBP1s expressing *vs* control hSAECs were retained (the DESEQ2 *P*_Adj_ < 0.05 was used as a cut-off to accommodate for multiple hypothesis-testing).

FXBP1s peaks were annotated to gene regions. Here we found that the 7086 peaks could be mapped by proximity to the regulatory regions or bodies of 4,827 unique genes. We found that the majority of FXBP1s binding peaks are found within gene bodies, introns and the proximal promoters (Figure 3C). To confirm the location of FXBP1s binding sites, a heat map was constructed relative to length-normalized genes. A strong peak in the proximal promoters was identified (Figure 3D); by contrast, a weaker association of binding over gene bodies was seen between the transcriptional start sites (TSS) and transcriptional end sites (TES) (Figure 3E).

To determine whether XBP1s-bound genes controlled specific cellular processes, a pathway enrichment was conducted for all genes with a FXBP1s peak within 3 kb of the transcription start site. We noted that the XBP1s binding genes controlled activity of the Rho GTPase cycle, diseases of signal transduction, Asparagine-linked glycosylation, and multiple ER-Golgi functions, consistent with XBP1s known role in mediating homeostatic response to ER stress (Figure 2F). A focused analysis of gene pathways controlled by FXBP1s binding to all genes with FXBP1s peaks within 1 kb of the promoter revealed similar major enrichments of Rho GTPase, signal transduction and N-linked glycosylation (Supplementary Figure S3).

### Enrichment of DNA-binding motifs

**Figure 4.**
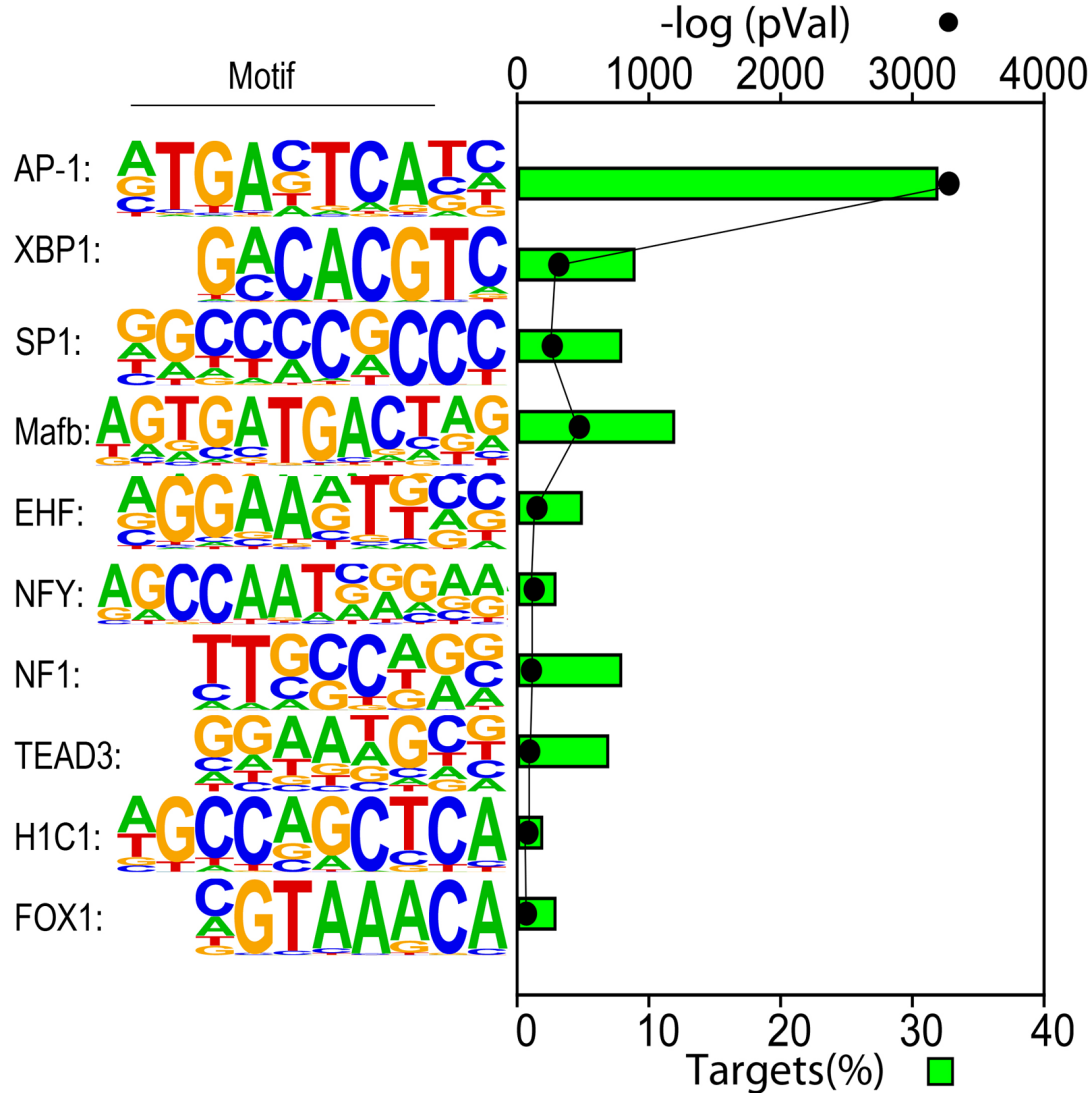
Binding sites enriched in XBP1s-bound DNA. *De novo* motif analysis of the 7000 FXBP1s bound sequences in the hSAEC genome. Motifs are rank ordered by the frequency of binding sites. For each, the p value of enrichment is also shown. Note that activator protein 1 (AP1) frequency is more frequent than the classic UPRE binding sites of XBP1s. The GC sequences corresponding to specificity protein 1 (SP1) binding sequences are also highly enriched. Despite searching for the TBP weight matrix, TATAA sequences are not significantly enriched in XBP1s binding sites. Abbreviations: EHF, ETS Homologous Factor; FOX1, forkhead box 1; HIC1, HIC ZBTB Transcriptional Repressor 1; Mafb, MAF BZIP transcription factor b; NFI, neurofibromin 1; NFY, nuclear transcription factor Y; TEAD, TEA Domain Transcription Factor 1.

Previous work has found that XBP1 binds to pleiotropic sequences, such as the UPRE and ERSE motifs. This variability is determined, in part, by heterotypic interactions with other bZIP DNA-binding proteins that are under cell-type, differentiation-state and stimulus-dependent control (14,15,30). To infer the binding sites enriched in FXBP1s-bound fragments in RSV-infected epithelial cells, we conducted motif enrichment analysis on the 7,086 high confidence FXBP1s peaks by scanning for 441 known transcription factor binding determining binding probability using position weight matrices (25). We observed enrichment of > 20 sequence motifs relative to background sequences (see Supplementary Figure S2). The top 10 ranking peaks identified included AP-1 motifs (containing the TGA(N)TCA core (30), present in 32% of total targets), XBP1 sequences (containing the CACGT core from JASPAR MA0844.1), SP1 sequences (containing the CCGCCC core) and others (Figure 4). Although TATA-box binding protein (TBP) binding was included in known search motif strategy, the only promoter consensus that was statistically enriched was the ‘GC-box’ SP1-binding sequence. We interpret these findings to suggest that FXBP1s interacts with DNA binding domains enriched in AP-1 motifs primarily in GC-box driven promoters.

### XBP1s binds superenhancers in gene bodies associated with rho-GTPase signaling

**Figure 5.**
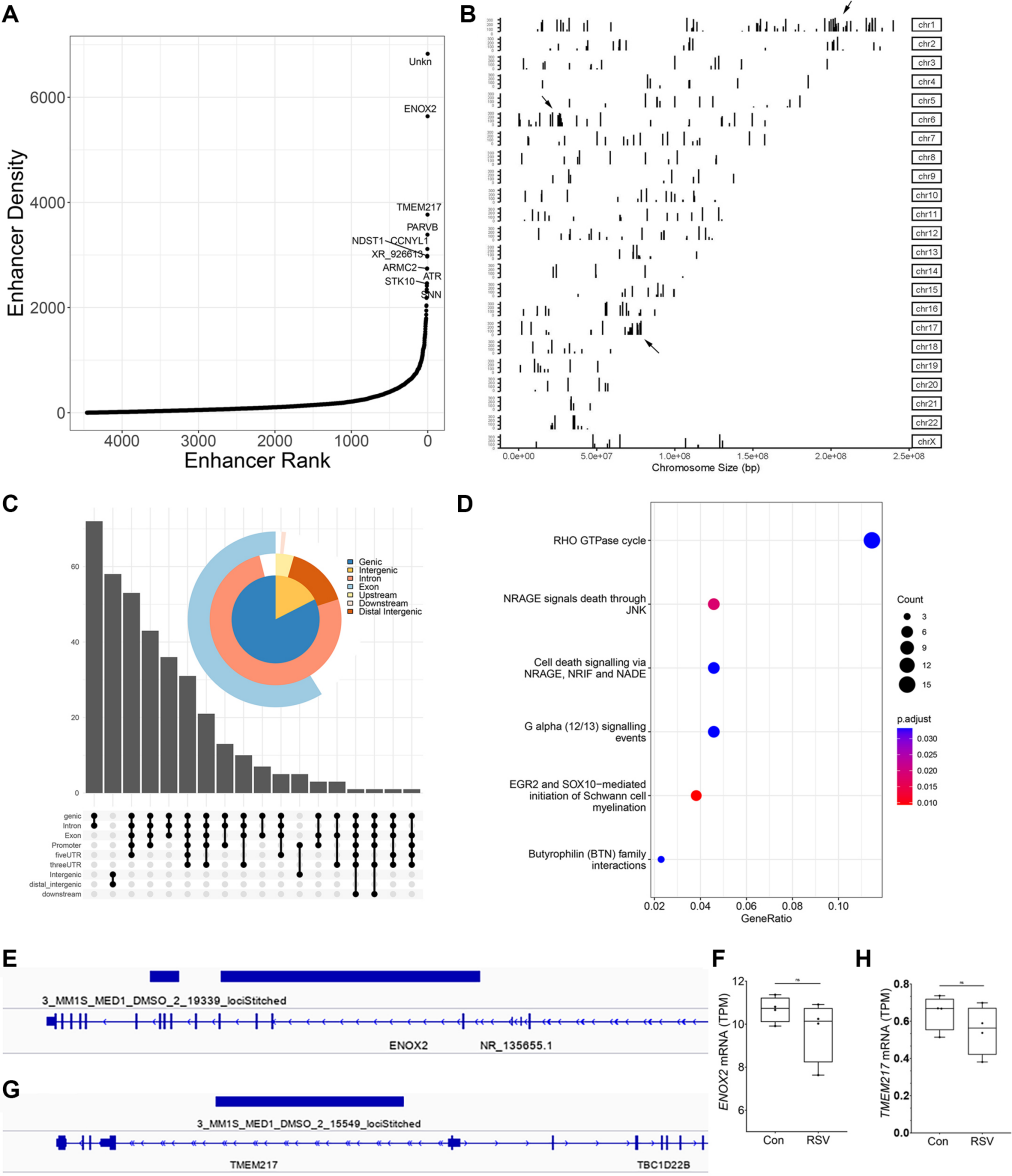
Analysis of XBP1s interactions with superenhancers. (**A**) Binding of FXBP1s to hSAEC Med1 superenhancers. The ROSE software (23) identified 362 superenhancers that were rank ordered by FXBP1s binding density; the top most enriched enhancers are identified by nearest gene. Abbreviations: ENOX2, Ecto-NOX Disulfide-Thiol Exchanger 2; TMEM217, transmembrane protein 217; PARVB, Parvin Beta; Unk, unknown gene region. (**B**) Chromosomal distribution of SE was determined for each chromosome. Shown is the location of superenhancers for each individual chromosome (Chr), displayed as a linear sequence. Several clusters of superenhancers are identified on Chr 1, 6, indicated by arrows. (**C**) Annotation of FXBP1s superenhancers on genes. Upset plot of FXBP1s-bound superenhancers was conducted. (**D**) Gene pathways of FXBP1s-bound superenhancers. Shown are genome ontologies ranked by the number of genes in a pathway (gene ratio) and by enrichment relative to genome (adjusted p value, padjust). (E–H) Expression of superenhancer-associated genes. Genes occupied by FXBP1s superenhancers was determined by RNA seq (16). (**E**) IGV view of superenhancer domain for ENOX2. (**F**) Expression of ENOX2. Plotted is transcripts per million (TPM) for mock or RSV infected cells in 25–75% box plot format; symbols are independent replicates. Ns, not significant. (**G**) IGV of TMEM217. (**H**) Expression of TMEM217.

Analysis of ChIP-Seq studies have revealed the existence of large clusters of regulatory elements >20 kb in length with substantially enriched binding of Med1, BRD4, or H3K27Ac coactivators, referred to as ‘superenhancers’ (23,32). To establish whether FXBP1s bound to genomic superenhancers, mapped FXBP1s peaks to Med1 superenhancers. We were able to identify 364 superenhancers in RSV infected hSAECs (Figure 5A). The location of these superenhancers were distributed across all chromosomes, with some superenhancer ‘clusters’ in Chromosome 1, 6 and 17 (Figure 5B). As with the gene body annotation, superenhancers were primarily in gene bodies and introns (Figure 5C). Functionally, these genes were enriched in Rho GTPase cycle in pathway analysis (Figure 5C), consistent with the earlier pathway analysis of all FXBP1s bound genes.

To initially explore expression of genes associated with XBP1-enriched superenhancers, we identified the two most highly enriched superenhancers associated with genes and examined their expression using our previous RNA-Seq study (16). One of the more highly enriched FXBP1s peaks lies within the Ecto-NOX Disulfide-Thiol Exchanger 2 (*ENOX2*) gene body (Figure 5E). We noted that *ENOX2* was expressed in uninfected hSAECs, and slightly, but not significantly, reduced in response to RSV infection. By contrast, the Transmembrane Protein 217 (*TMEM217*) gene was expressed at quite low levels in the RNA-Seq data, also being slightly reduced by FXBP1s interaction (Figure 5E). These data will require further exploration, but may suggest that FXBP1s destabilizes superenhancers in response to UPR-induced de-differentiation programs.

### XBP1s transactivates core HBP enzymes controlling UDP-GlcNAc synthesis

**Figure 6.**
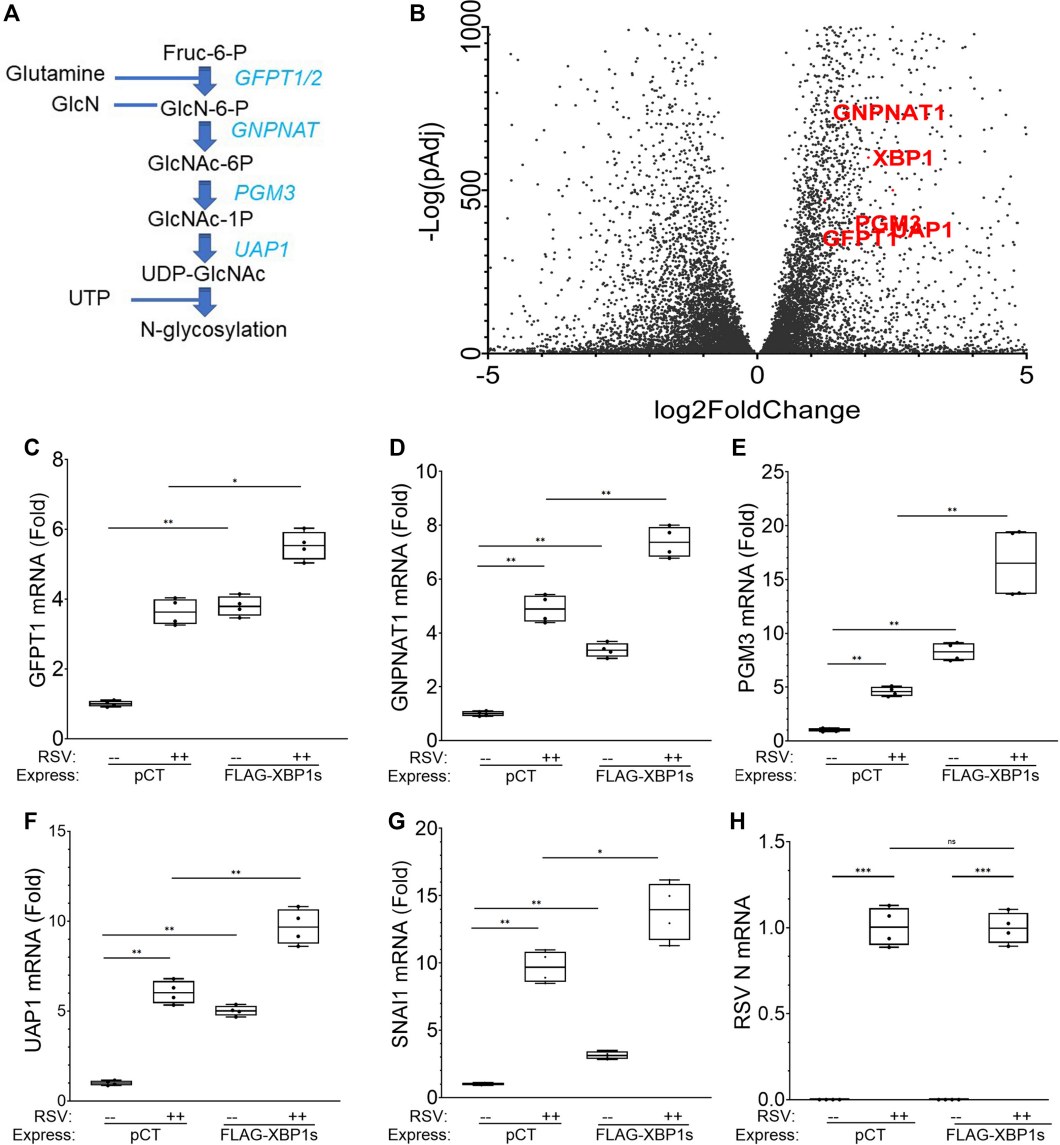
Coordinate activation of the core HBP pathway enzymes. **(A**) Enzymatic steps in hexosamine biosynthetic core pathway. Shown is a schematic of the HBP core and enzymatic conversion of D-fructose-6-phosphate (Fru-6-P) and l-glutamine to D-glucosamine-6-phosphate (GlcN-6-P) and l-glutamate. GlcN-6-P is an essential precursor of uridine 5′-diphosphate-N-acetyl-D-glucosamine (UDP-GlcNAc) required for protein N glycosylation. Abbreviations: GFPT1, Glutamine-Fructose-6-Phosphate Transaminase 1; GNPNAT1, Glucosamine-Phosphate N-Acetyltransferase 1; PGM3, Phosphoglucomutase 3; UAP1, UDP-N-Acetylglucosamine Pyrophosphorylase 1. (**B**) Volcano plot of RSV-induced gene expression. RNA seq analysis was examined for FXBP1s-binding genes. X axis, log2 fold change (TPM); Y axis, -log-transformed normalized pValue. Individual members of HBP are labeled in red and quantitated by Q-RT-PCR. (**C–G**) Q-RT-PCR validation of RSV and FXBP1s-regulated expression of HBP core pathway genes and the mesenchymal regulator SNAI1. hSAECs were transduced with empty (pCT) or FXBP1s-expressing (FXBP1s) lentivirus for 48 h at an MOI of 2.0. Cells were mock or RSV-infected (MOI = 1, 24 h). Shown is fold change of mRNA relative to mock infection as 25–75% box plots. Each symbol is an independent data point replicated in *n* = 4 independent experiments. (**H**) RSV transcription. Transcription of RSV N mRNAs is quantitated. Note that FXBP1s does not affect RSV transcription. **P < 0.01; ***P < 0.001; ns, not significant.

We next focused on the pathway genes bound by FXBP1s interacting at the proximal promoter. Here, a core of ER homeostasis genes was identified, including those controlling N-linked glycosylation, ER to Golgi transport (Figure 3F). To identify how RSV affected these genes, we analyzed an RNA-Seq data set of RSV-induced genes (16). Of significant interest to us, RSV significantly induces expression of *GFPT1, GFPT2, GNPNAT1, PGM3* and *UAP1*, comprising the five major core regulatory genes in the HBP pathway responsible for metabolizing fructose 6 phosphate into UDP-GlcNAc, (Figure 6A). Upregulation of these genes are indicated by volcano plot of RNA transcript abundance in mock-infected vs RSV-infected cells in Figure 6B.

We next independently validated the findings that RSV induces expression of HBP core regulatory enzymes by Q-RT-PCR. In this experiment, we also asked whether FXBP1s was sufficient to activate- or potentiate- their expression. For this, hSAECs were transduced with empty (pCT) or FXBP1s-expressing lentivirus (3d) and subsequently mock or RSV infected (MOI = 1.0, 24 h). In empty expression vector-transduced cells, we observed RSV infection increased *GFPT1* mRNA expression to 3.6 ± 0.4-fold, validating the RNA-Seq data (*P* < 0.0001 group-wise post-hoc; Figure 6C). Importantly, *GFPT1* expression was induced to a similar magnitude to 3.8 ± 0.29-fold by FXBP1s expression alone in the absence of RSV infection (*P* < 0.0001; Figure 6C). Moreover, *GFPT1* mRNA in FXBP1s transduced cells increased to 5.5 ± 0.4-fold after RSV infection (*P* < 0.01, Figure 6C). These data indicated that FXBP1s expression was sufficient to transactivate *GFPT1* expression, and its abundance was rate-limiting for *GFPT1* induction by RSV.

Similarly, expression of *GNPNAT1* was activated by RSV infection in empty expression vector-transduced cells to 4.9 ± 0.5-fold in response to RSV infection (*P* < 0.0001) and to 3.6 ± 0.3-fold by FXBP1s expression in mock-infected cells. The increase of *GNPNAT1* expression produced by RSV further increased with FXBP1s expression, in an additive fashion, where *GNPNAT1* mRNA expression increased to 7.4 ± 0.6-fold (*P* < 0.0001; Figure 6D). Remarkably, PGM3 expression was the most highly inducible of the HBP genes by FXBP1s transduction (by fold-change), with 8.28 ± 0.78-fold (*P* < 0.0001) induction by FXBP1s alone. The 4.6 ± 0.4-fold induction by RSV infection in empty vector-transduced cells was further potentiated by FXBP1s expression to as high as 16.5 ± 3.0-fold (*P* < 0.0001) induction (Figure 6E). A similar pattern, smaller in fold induction, of expression was observed for *UAP1* where FXBP1s expression was sufficient to transactivate the target gene in the absence of RSV infection and potentiated the effect of RSV (Figure 6F). As confirmation of our previous analysis, we observed FXBP1s potentiated the 9.7 ± 1.2-fold expression of the mesenchymal transcription factor, *SNAI1*, to 13.8 ± 2.2-fold (*P* < 0.0025, Figure 6G). Of note, the FXBP1s did not affect the level of RSV replication, measured by RSV nucleoprotein (N) expression, excluding the trivial possibility that potentiation of HBP core metabolic enzymes was due to an effect of enhancing RSV replication (Figure 6H). These data indicate that XBP1s is sufficient for transactivation of the HBP regulatory core enzymes.

### IRE1α-XBP1 is required for RSV-induced expression of the HBP core enzymatic machinery

**Figure 7.**
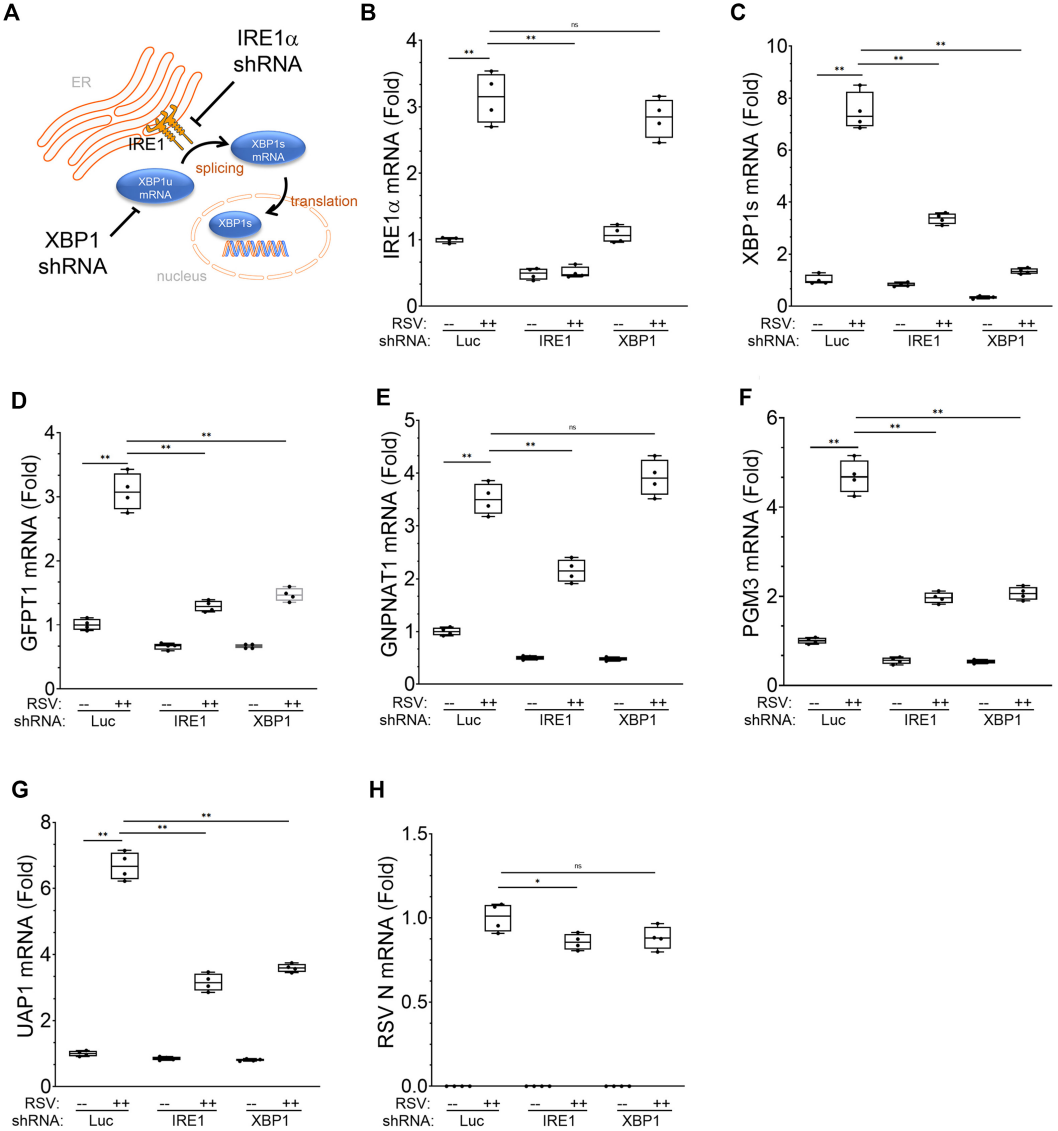
IRE1α-XBP1 signaling mediates RSV induced HBP activation. (**A**) Schematic of IRE1α-XBP1s activation and shRNA silencing. (B–H) Q-RT-PCR analysis of hSAECs stably expressing non-targeting shRNA (Luc), IRE1α-targeting shRNA (IRE1), or XBP1-targeting shRNA (XBP1). hSAECs were transduced with shRNA expression vectors for non-specific target (Luc), IRE1α- or XBP1-targeting. Shown are fold change of mRNA relative to mock infected hSAECs transduced with non-targeting shRNA (Luc). (**B**) Q-RT-PCR of *IRE1α* expression; (**C**) *XBP1s*; (**D**) *GFPT1*; (**E**) *GNPNAT1*; (**F**) *PGM3*; (**G**) *UAP1*; (**H**) RSV N. ns, not significant; *P < 0.05; **, *P* < 0.01, post-hoc.

Although RSV infection transactivates HBP enzymes and expression of FXBP1s is sufficient to activate the target genes, these data do not demonstrate that IRE1α-XBP1 pathway mediates the RSV effect. To study the role of IRE1α-XBP1s in RSV infection, *IRE1α* and *XBP1* were separately silenced using shRNA transduction (Figure 7A). We first analyzed *IRE1α* expression in hSAECs expressing non-targeting (Luc)-, IRE1α targeting-, or XBP1 targeting-shRNAs. We noted that IRE1α mRNA was significantly induced by RSV by 3.1 ± 0.4-fold relative to mock-infected controls (*P* < 0.0001; Figure 7B). By contrast, by expressing IRE1α-targeting shRNA, IRE1α mRNA was reduced to 50% that of non-targeting controls in mock-infected cells, and the RSV induction of IRE1α mRNA was completely inhibited (Figure 7B). In the hSAECs expressing XBP1-targeting shRNA, the level of IRE1α was unaffected relative to non-targeting shRNA transduced hSAECs (Figure 7B). These data indicate selective inhibition of IRE1α.

By contrast, RSV induced 7.5 ± 0.7-fold increase in *XBP1s* mRNA in non-targeting shRNA expressing cells; this effect was reduced by 54% in *IRE1α*-silenced cells, and 72% in *XBP1*-silenced cells (both *P* < 0.0001; Figure 7C). The dramatic reduction of XBP1s in IRE1α-silenced hSAECs confirms that IRE1α is upstream of XBP1s formation in RSV infection (Figure 7A).

We observed that *XBP1* silencing reduced basal *GFPT1* expression and RSV-induced *GFPT1* expression (3.1 ± 0.28 versus 1.3 ± 0.1-fold, *P* < 0.0001; Figure 7D). A similar finding was observed for *IRE1α* silencing, where both basal and RSV induced *GFPT1* expression were significantly reduced (Figure 7D). Quite surprisingly, we noted that the basal levels of *GNPNAT1* mRNA were significantly reduced by both IRE1 and XBP1 silencing and that RSV-induced *GNPNAT1* mRNA expression was significantly inhibited by *IRE1α* silencing (3.5 ± 0.3 versus 2.2 ± 0.2, *P* < 0.0001) but *XBP1* silencing had no effect (Figure 7E). We suspect the explanation is that IRE1α possesses both RNase and kinase activities, and through the UPRsome super-complex, the IRE1α kinase may elicit multiple signaling activities including IKK-NFκ6B and JNK-AP1 (33). In addition, *GNPNAT1* proximal promoter region is extraordinarily GC-rich containing as many as 8 conserved GC-box DNA motifs that can be recognized by zinc finger transcription factors, such as SP1, to provide compensatory *GNPNAT1* transactivation (Supplementary Figure S4B).

Furthermore, the effect of IRE1α and XBP1 silencing showed substantial inhibition of basal and RSV-induced expression of *PGM3* and *UAP1* (Figure 7F,G). Relative to non-targeting shRNA transduced cells, RSV replication in XBP1-targeted cells was 89% and that of IRE1α-targeted cells was 87%, which although significant, is not sufficient to account for the reduction in mRNA expression (Figure 7H). These data support the conclusion that IRE1α-XBP1s signaling is a major component of RSV-induced HBP expression.

### FXBP1s binds to proximal promoters of core HBP pathway genes

**Figure 8.**
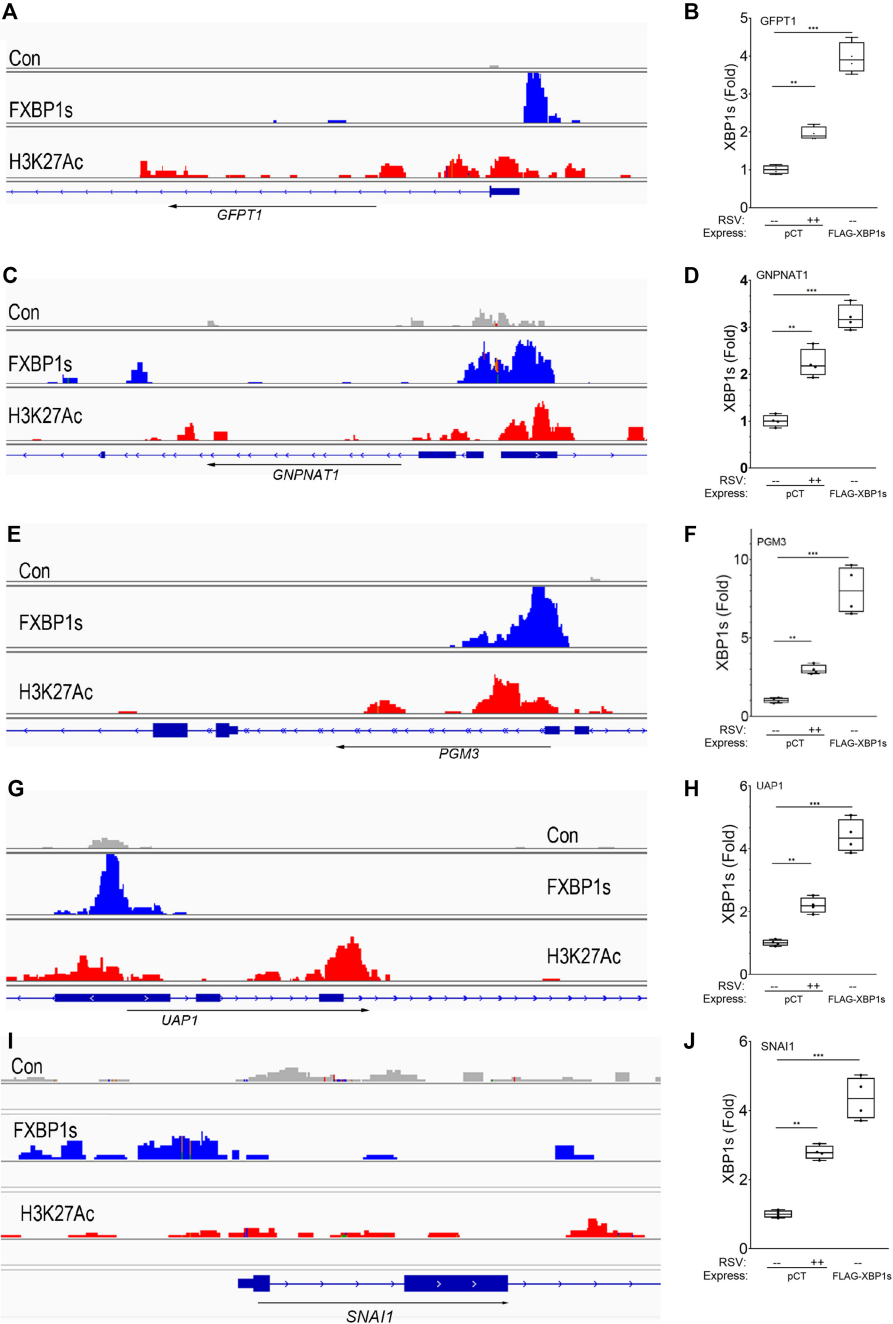
Validation of XBP1s binding to core HBP genes. Integrated genomics viewer (IGV) of the individual XBP1s peaks vs control cleavage patterns of HBP genes and *SNAI1* determined by CUT&RUN. Panels (**A**), (**C**), (**E**), (**G**), (**I**), RSV-induced XBP1s peaks on *GFPT1*, *GNPNAT1*, *PGM3*, *UAP1* and *SNAI1* respectively are indicated by the height of the blue peaks relative to the promoter of each gene. H3K27Ac peaks in RSV-infected hSAECs are shown. Arrow indicates direction of transcription. Panels (**B**), (**D**), (**F**), (**H**), (**J**), plot changes in XBP1s binding. XChIP was assayed in the absence (mock) or presence of RSV infection, or by FXBP1s expression (without RSV infection). Data are fold changes in Q-gPCR normalized to input and relative to mock infected controls. **, *P* < 0.01, post-hoc.

To better elucidate the role of XBP1s in transactivation of the HBP core enzymes, the profiles of FXBP1s binding to *GFPT1, GNPNAT1, PGM3*, *UAP1* and *SNAI1* genes were extracted from the CUT&RUN analysis and visualized on the 5′ regulatory elements and gene body using IGV viewer. In this visualization, we also aligned Lys-acetylated H3K27 (H3K27Ac) peaks determined in parallel CUT&RUN analysis; H3K27Ac marks were used as a measure of transcriptionally active chromatin (34). A sharp region of FXBP1s binding was observed for *GFPT1* proximal promoter (Figure 8A). We also noted that the *GFPT1* gene body lies with open chromatin domain with broadly distributed H3K27Ac peaks. To further confirm that XBP1s was contained within these peaks, XBP1s binding was measured using two-step chromatin immunoprecipitation assays (XChIP), a highly quantative ChIP method developed by us (19). We found that RSV induced a 1.9 ± 0.2-fold increase in XBP1s binding to *GFPT1* (*P* = 0.002; Figure 8B). Similarly, a 3.9 ± 0.4-fold increase in XBP1s binding was seen in cells transduced with FXBP1s expression vector (*P* < 0.0001; Figure 8B), confirming FXBP1s binds to the proximal *GFPT1* promoter in open chromatin.

Binding of FXBP1s was also observed for the *GNPNAT1* 5′ regulatory elements that corresponded to transcriptionally active H3K27Ac peaks (Figure 8C). By XChIP, XBP1s binding to the *GNPNAT1* promoter was increased 2.2 ± 0.3-fold relative to mock infected cells in response to RSV (*P* = 0.0001), and XBP1s binding increased to 3.2 ± 0.3-fold in FXBP1s-transduced hSAECs over mock-infected pCT transduced cells (*P* < 0.0001; Figure 8D). Similar patterns of inducible XBP1s and H3K27Ac peaks were observed for *PGM3*, *UAP1* and *SNAI1* promoters that were validated by XChIP (Figure 8-E, G, I). Specifically, in comparison to pCT-transduced mock-infected cells, FXBP1s expressing cells showed a significant increase in XBP1s binding to the promoters of PGM3, UAP1 and SNAI1 by 8.0 ± 1.5-fold (*P* < 0.0001), 4.4 ± 0.52-fold (*P* < 0.0001) and 4.6 ± 0.6-fold (*P* < 0.001), respectively (Figure 8F, H, J). These data directly validate that FXBP1s binds to the proximal promoters of mesenchymal regulatory genes and the HBP within transcriptionally active chromatin domains.

### Canonical UPR activators induce the HBP pathway

**Figure 9.**
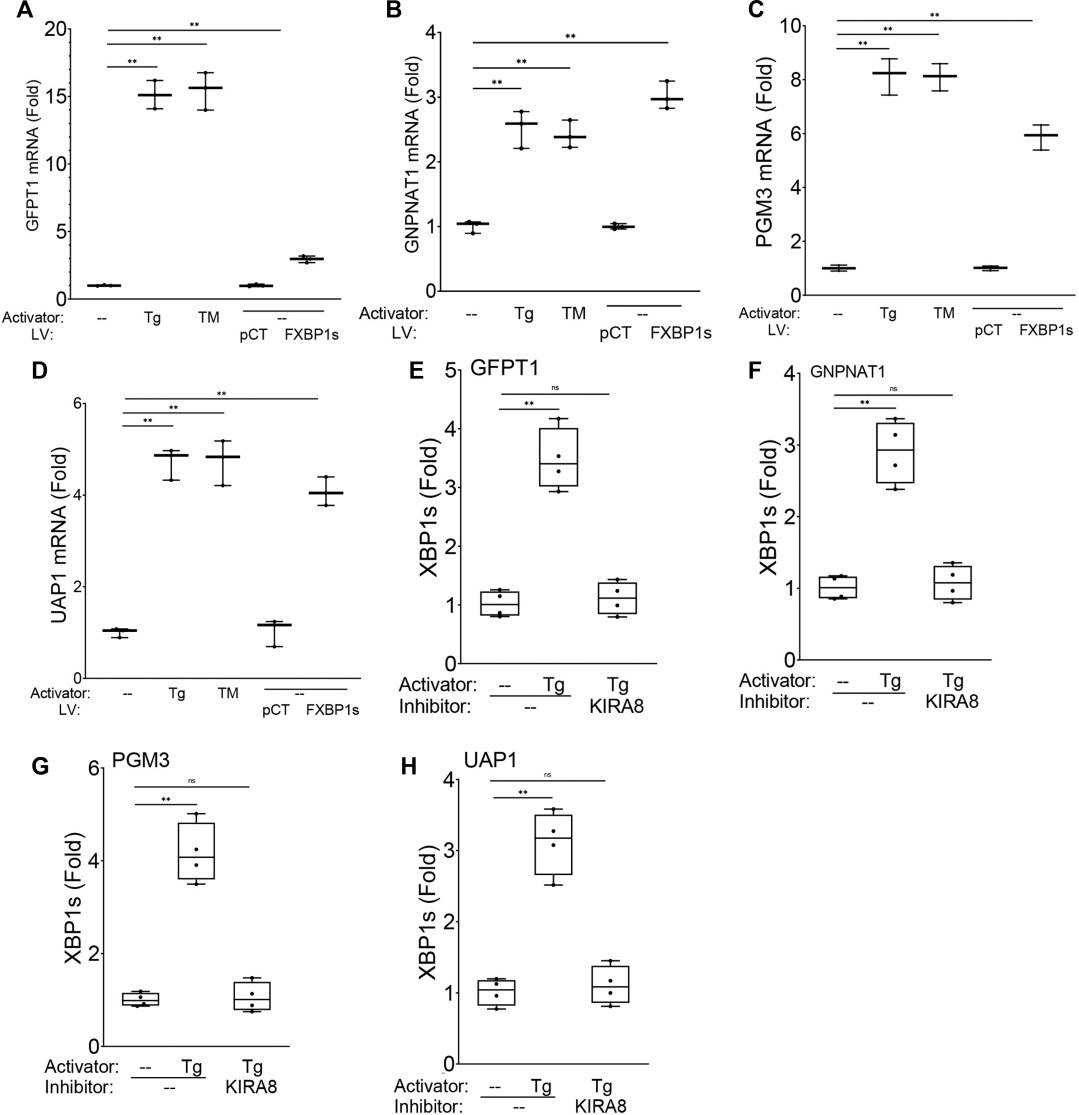
Canonical ER stress inducers directly activate the HBP pathway via IRE1α-XBP1s. To independently validate the finding that IRE1α-XBP1s pathway induces HBP core enzymes, hSAECs were treated with TM (0.5 ug/ml, 8 h), Tg (50 nM, 6 h) and compared with lentivirus transduction with empty (pCT) or FXBP1s-expression (MOI = 2, 48 h). Panels A-D, HBP mRNA levels were assayed by Q-RT-PCR. For each gene, shown is mean ± fold change of mRNA normalized to PPIA as internal control. (**A**), GFPT1; (**B**), GNPNAT1; (**C**), PGM3 and (**D**), UAP1.XChIP was conducted for the same genes after Tg treatment (50 nM, 6 h) in the absence or presence of KIRA8 (10 μM). Shown is fold-change in XBP1s binding normalized to input and relative to mock treated cells. Panels (**E**) *GFPT1*; (**F**) *GNPNAT1*; (**G**) *PGM3* (**H**) *UAP1*. **P < 0.001, post-hoc.

To independently validate the findings that HBP gene pathway was activated by UPR, we conducted experiments activating the UPR using ER stress inducers. In this series of experiments, hSAECs were treated with optimized concentrations of TM (0.5 μg/ml, 8 h) or Tg (50 nM, 6 h) (Supplementary Figure S1) and Q-RT-PCR assays were conducted to determine HBP core pathway activation. We observed that TM treatment increased *GFPT1* mRNA 15.1 ± 1-fold relative to solvent-treated controls (*P* < 0.0001), and that Tg induced *GFPT1* mRNA to 15.5 ± 1.4-fold (*P* < 0.0001; Figure 9A). FXBP1s expression induced *GFPT1* to a lesser degree by 3.0 ± 0.2-fold (Figure 9A). Similarly, TM increased *GNPNAT1* by 2.5 ± 0.3-fold, and Tg induced *GNPNAT1* by 2.4 ± 0.2-fold (both *P* < 0.0001, Figure 9B); whereas FXBP1s activated *GNPNAT1* by 3.1 ± 0.2 -fold (*P* < 0.001, Figure 9B). We found that *PGM3* was induced by 8.1 ± 0.7-fold by TM, 8.1 ± 0.5 -fold by Tg and 5.9 ± 0.5 -fold by FXBP1s expression (*P* < 0.0001; Figure 9C). Finally, TM induced *UAP1* expression by 4.7 ± 0.3-fold, and Tg induced it by 4.7 ± 0.5-fold. FXBP1s expression increased *UAP1* by 4.1 ± 0.32-fold (all *P* < 0.001, Figure 9D). Collectively these data indicated that: (i) activation of the UPR by canonical activators of ER stress activate the HBP pathway; and (ii) FXBP1s activates the same genes, although in some cases less efficiently, suggesting that endogenous activation of ER stress also induces additional signaling cascades enhancing HBP expression.

### Endogenous XBP1s is recruited by HBP pathway genes

To confirm that HBP genes were binding targets of endogenous IRE1α-XBP1s pathway, we conducted XBP1s XChIP analyses after activation of ER stress by Tg treatment in the absence or presence of the IRE1α RNase inhibitor, KIRA8. We observed that Tg induced a 3.5 ± 0.5-fold increase in endogenous XBP1s binding to the *GFPT1* promoter (*P* < 0.0001; Figure 9E). This induction was completely inhibited by the treatment with KIRA8 (Figure 9E). We found that Tg induced a 2.9 ± 0.4-fold increase in XBP1s binding to the *GNPNAT1* promoter (*P* < 0.0001; Figure 9F), also in a manner dependent on IRE1, as this induction was inhibited by KIRA8 (Figure 9F). Tg inducedd a 4.2 ± 0.6-fold increase in XBP1s binding to the *PGM3* promoter, and a 3.1 ± 0.5-fold increase in binding to the *UAP1* promoter (both *P* < 0.0001; Figure 9G, H, respectively). In all cases, the treatment with the IRE1α RNase inhibitor blocked XBP1s binding, indicating that the endogenous IRE1α-XBP1s pathway directly activated the HBP genes.

### FXBP1s binding recruits phospho-ser2 CTD RNA pol II binding to HBP core genes

**Figure 10.**
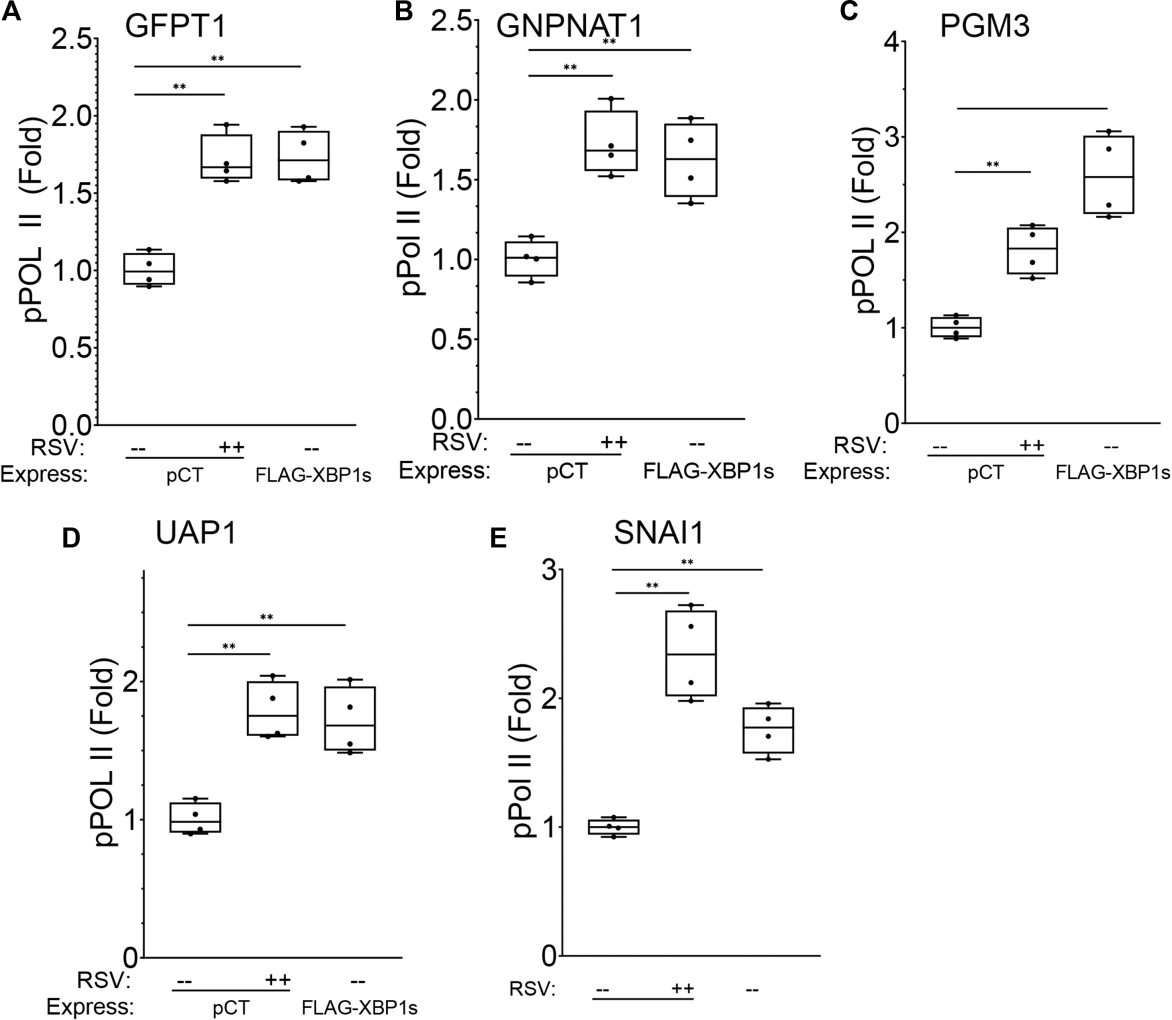
RSV infection and XBP1s binding induce phospho-Ser 2 CTD Pol II binding to the core HBP pathway genes. Recruitment of pSer2-Pol II in response to RSV or in response to FXBP1s expression was determined by XChIP using pSer2-Pol II -specific antibody. Shown is fold change in pSer2-Pol II binding normalized to input and relative to mock infected non-targeted control. (**A**) *GFPT1*; (**B**) *GNPNAT1*; (**C**) *PGM3*; (**D**) *UAP1*; (**E**) *SNAI1*. Data are fold changes in Q-gPCR normalized to input and relative to mock infected controls. **P < 0.01, post-hoc Tukeys.

To better understand how FXBP1s binding facilitates gene expression, we tested whether FXBP1s may contribute to transcriptional elongation. Transcriptional elongation is the primary regulated step of immediate early response gene induction. Mechanistically, the hallmark of transcriptional elongation is the recruitment of Ser2 phosphorylated RNA Pol II (pSer2-Pol II) to a proximal promoter (35,36). We asked whether either RSV infection or FXBP1s binding induced pSer2-Pol II binding to the HBP proximal promoters. We found that RSV infection induces a 1.7 ± 0.6-fold increase in pSer2-Pol II binding to the *GFPT1* promoter (*P* = 0.0002; Figure 10A). Interestingly to us, FXBP1s was sufficient to also induce a 1.7 ± 0.2 increase in pSer2-Pol II binding to GFPT1 (*P* < 0.0001; Figure 10A). Similarly, pSer2-Pol II binding to the promoters of *GNPNAT1*, *PGM3*, *UAP1* and *SNAI1* was induced by RSV by 1.72 ± 0.2-fold (*P* < 0.01), 1.81 ± 0.26-fold (*P* < 0.01), 1.79 ± 0.21-fold (*P* < 0.01) and 2.3 ± 0.4-fold (*P* < 0.0001), respectively (Figure 10B-E). FXBP1s induced a 1.6 ± 0.2 (*P* < 0.01), 2.6 ± 0.4 (*P* < 0.01), 1.7 ± 0.2 (*P* < 0.01) and 1.8 ± 0.2-fold (*P* < 0.01) -fold increase in pSer2-Pol II binding to the *GNPNAT1*, *PGM3, UAP1* and *SNAI1* promoters, respectively (Figure 10B-E). Collectively, these data indicate to us that *GFPT1, PGM3*, *UAP1* and *SNAI1* contain proximal promoter XBP1s binding sites whose recruitment results in pSer2-Pol II recruitment and gene activation.

### XBP1 is required for pSer2-pol II recruitment

**Figure 11.**
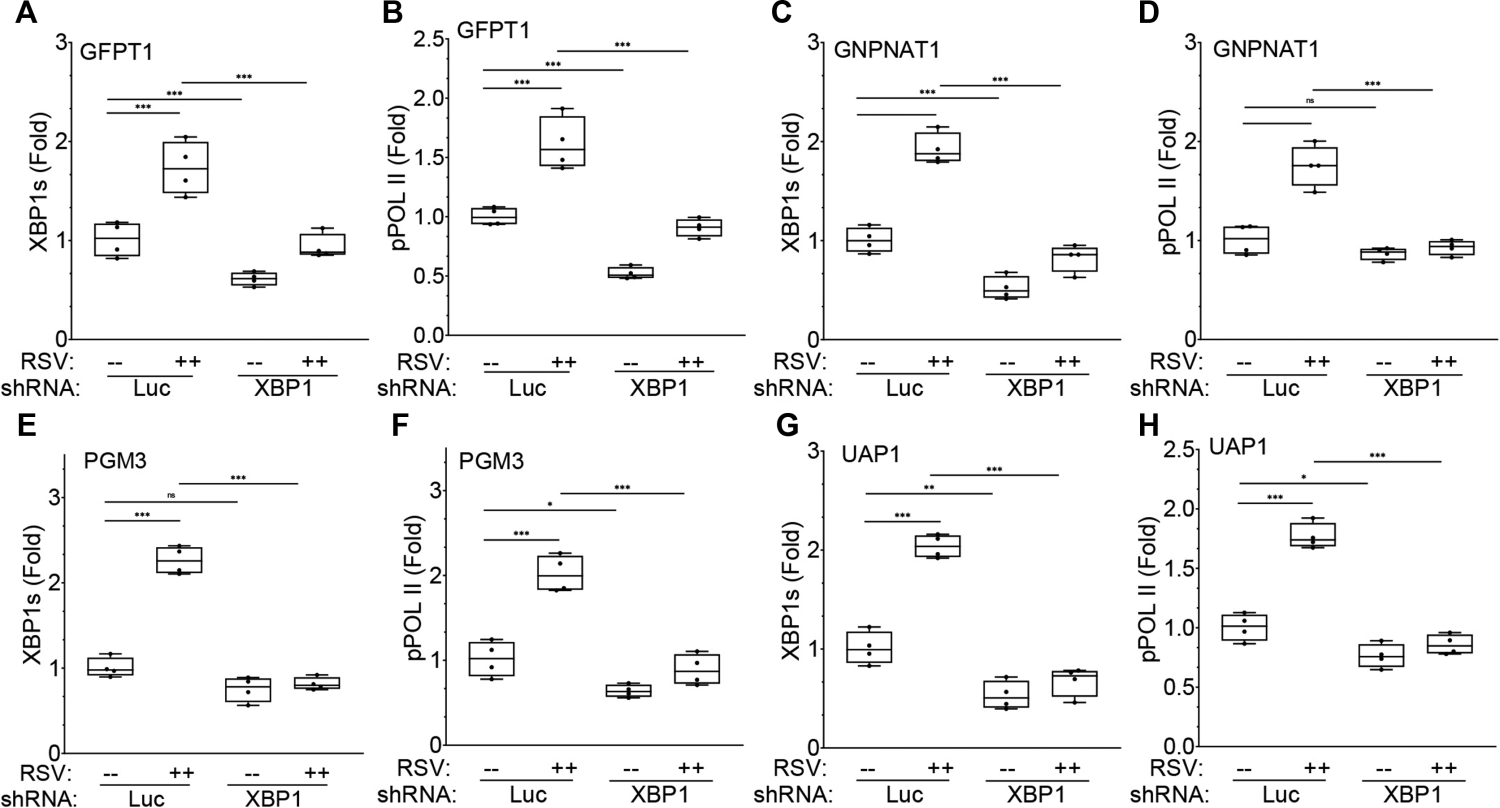
IRE1α-XBP1s is required for pSer2-Pol II binding to core HBP genes. The effect of XBP1 silencing on RSV-induced binding of XBP1s and pSer2-Pol II (pPol II) to HBP promoters was determined by XChIP. Shown is fold change in transcription factor binding normalized to input and relative to mock infected non-targeted control. (A, B) effect of knockdown on binding to *GFPT1* promoter. (**A**) XBP1s binding; (**B**) pPol II binding. (**C, D**) Effect of knockdown on binding to *GNPNAT1* promoter. (**C**) XBP1s binding; (**D**) pPol II binding. (E, F) Effect of knockdown on binding to *PGM3* promoter. (**E**) XBP1s binding; (**F**) pPol II binding. (**G, H**) Effect of knockdown on binding to *UAP1* promoter. (**G**) XBP1s binding; (**H**) pPol II binding. *P < 0.05; **P < 0.01; ***P < 0.001, post-hoc Tukeys. ns, not significant.

We next tested whether XBP1s binding was required for RSV-induced recruitment of pSer2-Pol II recruitment. In this experiment, mock- or RSV-infected non-targeting shRNA transduced or XBP1-silenced hSAECs were subjected to XChIP. We first measured XBP1s binding by XChIP to confirm functionally significant XBP1 depletion. We observed that RSV induced a 1.7 ± 0.3-fold increase of XBP1s binding to *GFPT1* promoter in hSAECs expressing non-targeting shRNA; by contrast, XBP1s binding to *GFPT1* was reduced by 61% in hSAECs expressing XBP1 shRNA in response to RSV infection (Figure 11A). These data confirm that XBP1 shRNA sufficiently depleted XBP1 to reduce its inducible recruitment to *GFPT1*. We next examined whether reduction in XBP1s binding affected recruitment of pSer2-Pol II to the promoter by XChIP. We found that in non-targeting shRNA-transduced cells, RSV increased pSer2-Pol II binding by 1.6 ± 0.2-fold to the *GFPT1* promoter, and in the absence of RSV-induced XBP1s binding, pSer2- Pol II binding was reduced to less than that of mock-infected cells (0.9 ± 0.07-fold, *P* < 0.0001, Figure 11B). These data demonstrate that XBP1s binding was required for pSer2-Pol II recruitment.

Similar findings were observed for *GNPNAT1* promoter recruitment of pSer2-Pol II, where RSV induced a 1.9 ± 0.2-fold increase of XBP1s binding in hSAECs transduced with non-targeting shRNA, and it was reduced to below that of Mock-infected cells in those transduced with XBP1 shRNA (Figure 11C), indicating again efficient gene knockdown. In non-targeting shRNA transductants, RSV induced a 1.8 ± 0.2-fold increase of pSer2- Pol II binding that was reduced to control levels by XBP1 silencing (0.9 ± 0.1-fold, *P* < 0.0001, Figure 11D). Similar patterns were seen for XBP1s and pSer2-Pol II binding for the *PGM3* and *UAP1* promoters in response to RSV infection and *XBP1* silencing (Figure 11E–H). Together these results indicate that XBP1s binding mediates transcriptional elongation through recruitment of pSer2-Pol II binding to the HBP pathway genes.

## DISCUSSION

EMP is a coordinate cellular response to mucosal injury that plays a key role in tissue repair. Unchecked, EMP is associated with excessive ECM deposition, fibrosis and organ dysfunction. Although EMP is orchestrated by interactions of the IKK signaling with multiple intracellular phosphorylation cascades, maintenance of the EMT pathway is dependent on cellular survival and adaptation to ER stress through protein N-glycosylation (13,37). EMP-induced N-glycosylation is dependent on the abundance of intracellular uridine 5′-diphosphate-N-acetyl-d-glucosamine (UDP-GlcNAc), which is a rate-limiting substrate for N-glycan synthesis and subsequently N-glycosylation of asparagines on secreted proteins, enabling proper protein folding, transport to Golgi and secretion, relieving proteotoxicity (13,37). The details of how IRE1α-XBP1s pathway controls UPR-induced metabolic adaptations are incompletely understood. In this study, we dissect the mechanism how the UPR activates HBP through the IRE1α-XBP1s pathway using highly sensitive CUT&RUN approach enabled by FXBP1s expression in the setting of IKK activation, a situation that stabilizes nuclear FXBP1s (5). Our conclusions about the presence of direct XBP1s binding sites are strengthened by the use of the FLAG affinity tag, expressed at physiologically relevant levels, and whose isolation using anti-FLAG excludes potential confounding XBP1u signals, a limitation of prior work (15). Remarkably of the 4,780 genes directly bound XBP1s, a significant fraction are within 1 kb of the TSS. We further demonstrate that these genomic sequences are enriched in AP-1 binding and GC-box sequences, typical of ‘TATA-less’ promoters. Gene silencing experiments provide the first demonstration, to our knowledge, that XBP1s recruits processive-competent pSer2-Pol II, activating the HBP by coordinate transcriptional elongation.

Activation of the UPR involves expression of immediate-early homeostatic genes that prevent proteotoxicity via protein N-glycosylation (13,37). Our previous ATAC-Seq studies have shown that the majority of RSV-inducible genes are silent, yet maintained in an open chromatin configuration associated with H3K27Ac marks (16,29). H3K27Ac marks are characteristic of transcriptionally active chromatin. Here, immediate-early innate genes are engaged with hypo-phosphorylated RNA Pol II, a state that results in the production of short, unspliced transcripts (28,38). The recruitment of transcriptional elongation complexes, such as PTEF containing cyclin dependent kinases, results in Ser 2 phosphorylation in the heptad repeats of the Pol II carboxyterminal domain, a post-translational modification that licenses the paused RNA Pol II to become fully processive, enabling transcription of full-length mRNAs (39). Our discovery that XBP1s binding is necessary and sufficient for recruiting the processive pSer2-Pol II indicates that XBP1s is a trigger for transcriptional elongation of core HBP complex genes. This mechanism may be through direct interactions of XBP1s with the transcriptional elongation kinases, or removing negative elongation factors from paused promoters. More work will be required to understand how XBP1s recruits transcription elongation complexes and Pol II directed kinases to UPR-inducible immediate-early genes.

Work of others have shown that the IRE1α-XBP1s pathway plays an important role in B- and T lymphocyte maturation and differentiation, important in supporting secretion of immune-modulators by these highly specialized protein-secreting cells (15,40). In contrast, this study focuses on the role of IRE1α-XBP1s in epithelial cells as they de-differentiate by the EMP (16,41,42). Here, EMP is a potent inducer of cytokine, interferon, extracellular matrix and defensin secretion (13,43). As differentiated epithelial cells are non-secretory cells, this transition represents a major proteotoxic stress triggering metabolic adaptation.

**Figure 12.**
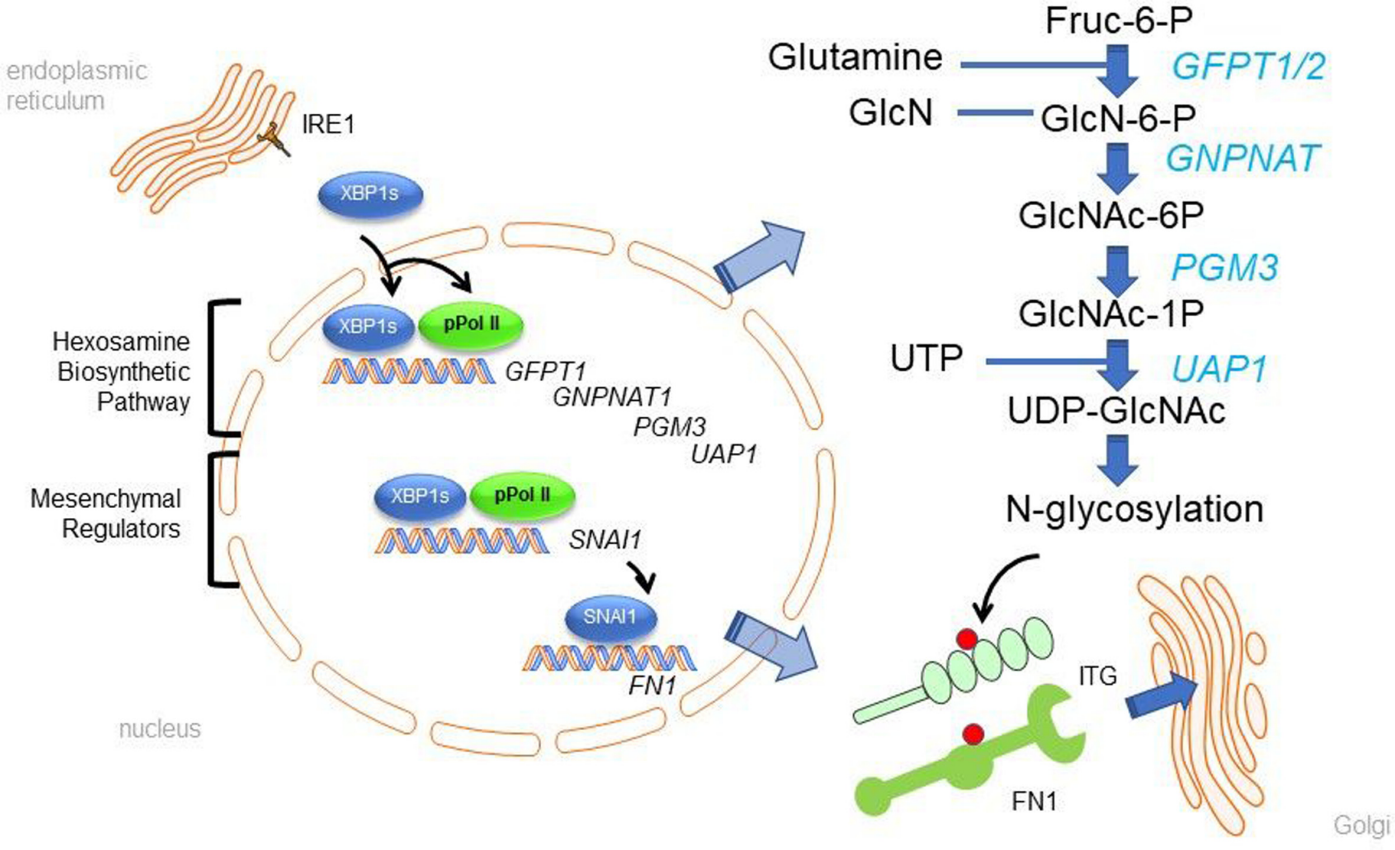
Graphical summary. Schematic of promoter recruitment mechanism for RSV induced EMP/HBP expression. IRE1α activation in the ER produces XBP1s mRNA that translates into XBP1s protein binding to high affinity genomic binding sites. XBP1s binding recruits pSer2-Pol II to TATA-less mesenchymal transcription factors and HBP pathway genes resulting in full length mRNA expression. Upon translation, the RSV-infected cell undergoes metabolic transition to synthesis of UDP-GlcNAc promoting N-glycosylation supporting epithelial mesenchymal plasticity and survival from proteotoxicity.

Here, we extend the understanding of EMP-induced metabolic reprogramming to show this process is mediated by the IRE1α-XBP1s pathway, coordinately expressing EMP and the HBP core enzymes (Figure 12). Expression of the SNAI1 mesenchymal regulator activates ECM expression. To ensure proper post-translational folding the HBP is coordinately activated. The HBP is a defined metabolic pathway that shunts up to 2% of intracellular glucose pool into UDP-GlcNAc in a series of 5 enzymatic steps. The initial, rate-limiting conversion is mediated by GFPT1 converting D-fructose-6-phosphate (Fru-6-P) and l-glutamine to D-glucosamine-6-phosphate (GlcN-6-P). GNPNAT1 catalyzes Ac-CoA and GlcN-6-P to generate N-acetylglucosamine-6-phosphate (GlcNAc-6P) and CoA. PGM3 isomerizes GlcNAc-6P into GlcNAc-1-phosphate (GlcNAc-1-P). Finally, UAP combines UTP and GlcNAc-1P to produce UDP-GlcNAc. A body of work, including our own have shown that UDP-GlcNAc is the rate-limiting substrate for protein N- and O-linked glycosylation (44). Our systems-level proteomics studies have shown that the upregulation of protein N glycosylation is critical to supporting secretion of structural components of the basal lamina and secreted ECM (13,37). In these studies, we found the use of selective small molecule inhibitors of the IRE1-XBP1s pathway reduce UDP-GlcNAc formation and secretion of N-glycosylated integrins, Laminins and Fibronectin, reducing ECM formation. The studies in this report clearly demonstrate that HBP core enzymatic pathway is transactivated by FXBP1 based on the observations that: (i) FXBP1s expression is sufficient to induce expression of sequential enzymes in UDP-GlcNAc synthesis; (ii) XBP1s inducibly binds to proximal promoters and (iii) silencing XBP1s blocks RSV-induced gene expression and pSer2-Pol II recruitment.

DNA binding and PCR selection studies seeking to define XBP1 binding motifs have found that XBP1s binds to highly pleiotropic DNA sequences. These DNA-recognition sites are influenced by cell-type and differentiation state, perhaps through induction of heterodimeric bZIP partners, such as ATF6 or NFY, that affect sequence recognition (18,30). Our transactivation studies indicate that, in RSV-infected epithelial cells, XBP1s can activate distinct UPRE and ESRE sequences. Although these sequences are well-established, our motif enrichment studies may indicate additional heterogeneity in XBP1 recognition sites. One interpretation of the striking enrichment of AP-1 sequences in FXBP1s-bound chromatin peaks is that XBP1s, or an XBP1s-heterodimer may directly interact with AP-1 sequences in the setting of RSV infection. To this point, others have reported that XBP1s binds to an AP-1/CRE element in the regulation of brain natriuretic peptide in cardiomyocytes (45). Whether this binding was of the XBP1 homodimer or a heterodimeric sequence has not been addressed. We also recognize that another interpretation is that XBP1s cooperates with adjacent AP-1-bound enhancers in target gene expression. More work will be required to understand how distinct protein interactions influence XBP1 genomic targeting.

We also note the marked enrichment of SP1/GC-box motifs in the XBP1s-binding peaks. SP1 is a transcription factor that mediates chromatin remodeling and gene regulation on TATA-less gene promoters (46). Sequence inspection of the promoters of GFPT1, GNPNAT1, PGM3, UAP1 and SNAI1 shows that all of these promoters are TATA-less with variable numbers of GC box sequences clustered around the transcriptional start site (Supplementary Figure S4). Noted earlier, the most highly inducible *PGM3* promoter contains more highly clustered GC boxes than other genes studied. The mechanism how XBP1s activates transcriptional elongation in GC-enriched gene promoters will require further investigation.

A striking finding of our analysis of FXBP1 peaks is the relative paucity of interactions with superenhancers. Of the 7, 086 FXBP1 sites identified in our CUT&RUN analysis, only 364 superenhancers were found. Our definition of superenhancer used generally accepted characteristics of 1. a high density of FXBP1s binding; 2. >10 kb in length; and, 3. regions that correspond to H3K27Ac- and Med1 rich binding regions (23). Although the earlier studies previously identified superenhancers to be important in controlling genes determining cell-type identity in stem cells, the genes associated with FXBP1s-enriched superenhancers in our data are expressed at variable levels, and not associated with epithelial differentiation programs. Whether XBP1s destabilizes superenhancers to promote genomic plasticity will require further studies.

In this manuscript, we focus on the effect of RSV on EMP and metabolic adaptation. RSV is a ubiquitous paramyxovirus of the Human Orthopneumovirus family that is a major cause of pediatric infections and hospitalizations in Western countries (47). In addition to its acute morbidities of pneumonia and hypoxia, severe RSV infections reshape the pulmonary immune response, resulting in Th2 polarization, produce airway remodeling, and allergic sensitization (48). Our RNA seq studies clearly demonstrate the presence of EMP, with activation of the SNAI1-Zeb induced EMT program, as well as activation of SMAD7-induced MET program. In addition, RSV is a potent inducer of ECM deposition and remodeling through SNAI1-driven fibronectin secretion and NFκB-dependent MMP9 production. These findings potentially link EMP with pathology associated with RSV infection. Long-term observational studies demonstrate RSV infections are associated with reduced lung function (49).

The mechanisms how RSV infections produce remodeling and impaired lung function are incompletely understood. In this study, we advance the understanding how RSV induces airway remodeling by providing direct mechanistic insights into the relationship between RSV-induced EMP, ER stress, and activation of the HBP pathway. The enrichment of promoters bound and regulated by XBP1s for genes controlling glycosylation indicates that the HBP is a major regulatory network of RSV-induced UPR signaling. Our data suggest that RSV may drive airway remodeling through metabolic adaptations from the EMP. Enhanced protein N-glycosylation supports ECM deposition, modifying the basal lamina of the airway.

In summary, we advance the understanding of the IRE1α-XBP1s pathway in metabolic adaptation to virus induced epithelial plasticity focusing on the HBP. The HBP is activated in response to TGFβ or viral infection, and provides homeostatic signals to increase the cellular capacity for protein N-glycosylation to reduce proteostasis. A remarkable finding from this work is the enrichment of proximal promoter binding sites recognized by XBP1s. Mechanistic studies of the consequences of XBP1s binding to the core HBP genes, we suggest that XBP1s acts to promote transcriptional elongation on SP1-enriched promoters by recruitment of processive RNA Pol II. Activation of this cellular stress response enhances ECM secretion, providing novel insights into how RSV infections can be associated with long term airway remodeling.

## DATA AVAILABILITY

The datasets presented in this study can be found in online repositories. The RNA seq is from GSE161849 at https://www.ncbi.nlm.nih.gov/genbank. CUT&RUN data is deposited under GEO GSE214786 is available on Jan 1, 2023 at https://www.ncbi.nlm.nih.gov/geo/query/acc.cgi?acc=GSE214786.

## Supplementary Material

gkad077_Supplemental_File
