# Supplementary material for: Genomic targets of the IRE1-XBP1s pathway in mediating metabolic adaptation in epithelial plasticity
Source: Nucleic Acids Res. 2023 Feb 11;51(8):3650–70. doi: 10.1093/nar/gkad077 (PMC10164557; doi:10.1093/nar/gkad077)

**Supplementary Figures**

**Supplementary Figure S1.**  Dose-finding experiments of the UPR in response to TM or Tg. hSAECs were treated with 0.5, 1.0 or 2.5 μg/ml TM or 50 nM Tg for different time period as indicated. XBP1s mRNA levels were assayed by Q-RT-PCR, with PPIA as internal control. For comparison, empty lentivirus (pCT) and FXBP1s expression (MOI=2) is shown. Note that XBP1s production peaked in response to 0.5 ug/ml TM after 8 h and that Tg peaked after 6 h.

**Supplementary Figure S2.** *De novo* motifs in FXBP1 enriched sequences. Show are the top 14 de novo motifs identified by position weight matrix sequence comparison (HOMER software). For each sequence, the P-value of enrichment, log-transformed P value, the percentage of targets in the FXBP1 sequences, the percentage of motifs identified in background and motif details.

**Supplementary Figure S3.** Gene pathways controlled by FXBP1s-bound genes within 1 kb of the transcription start site. Shown are genome ontologies ranked by the number of genes in a pathway (gene ratio) and by enrichment relative to genome (adjusted p value, padjust). In addition to cellular signaling, note the multiple entries for protein N glycosylation, ER processing and ER-Golgi transport. These pathways are shared with the genes bound by FXBP1 within 3 kb of the transcription start site.

**Supplementary Figure S4.** Location of regulatory motifs in HBP gene promoters. Shown are FXBP1 motifs extracted from CUT&RUN. XBP1 GAC sequences and GC boxes are shown, along with the location of transcriptional start sites (TSS). (**A**), GFPT1 promoter; (**B**) GNPNAT1 promoter; (**C**) PGM3 promoter; (**D**) UAP1 promoter; (**E**) SNAI1 promoter. Note the absence of TATAA box sequences are replaced by GC boxes.

**Supplementary Figure S1.**

**
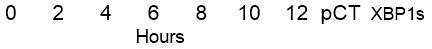

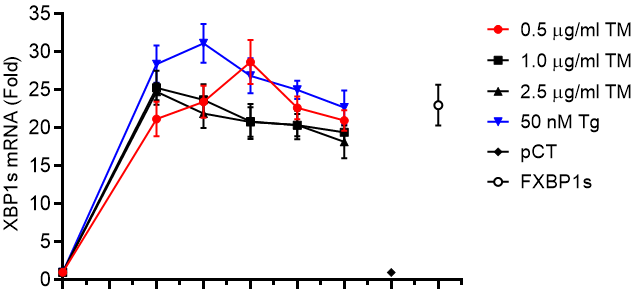
**

**Supplementary Figure S2**


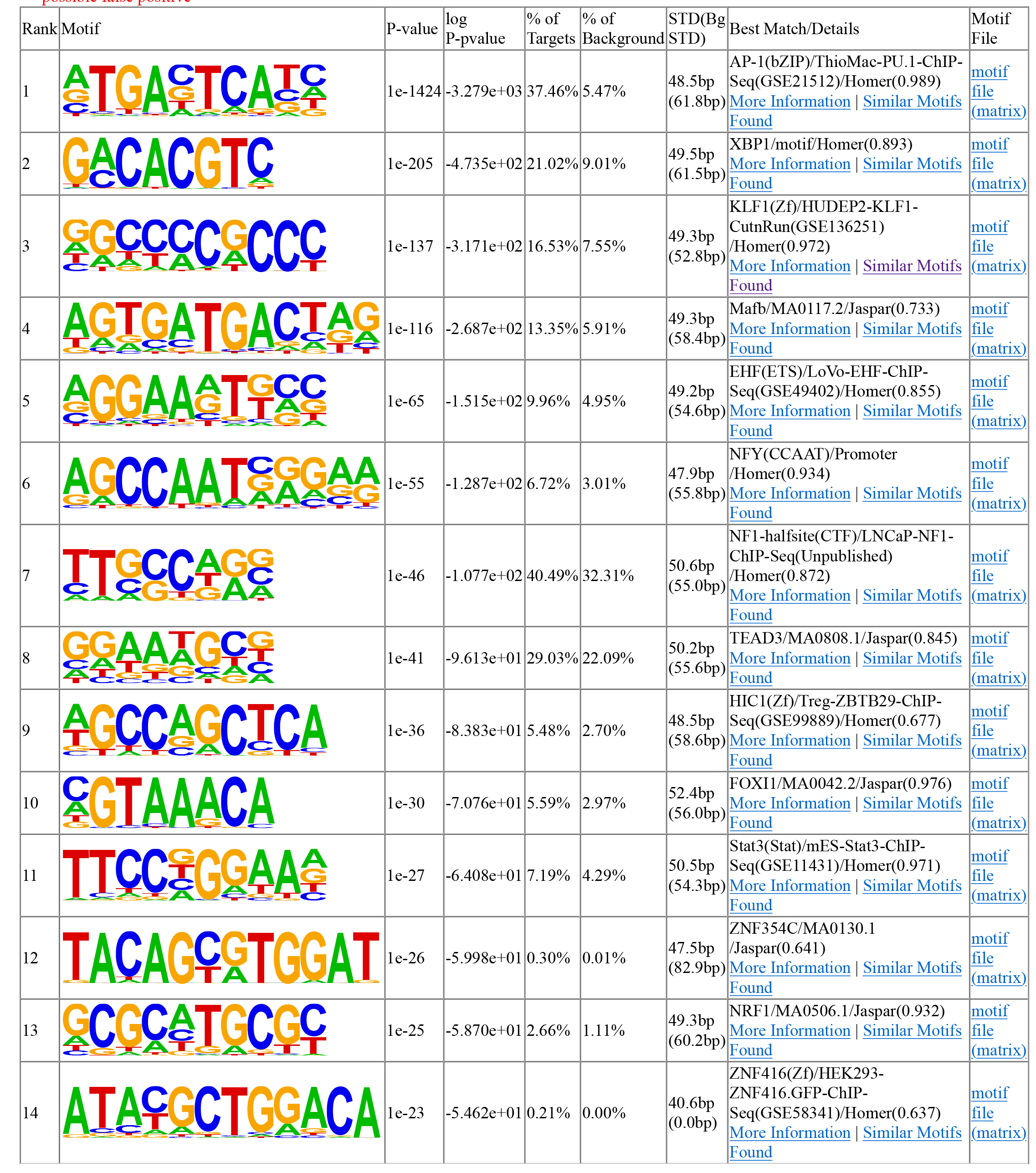


**Supplementary Figure S3.**


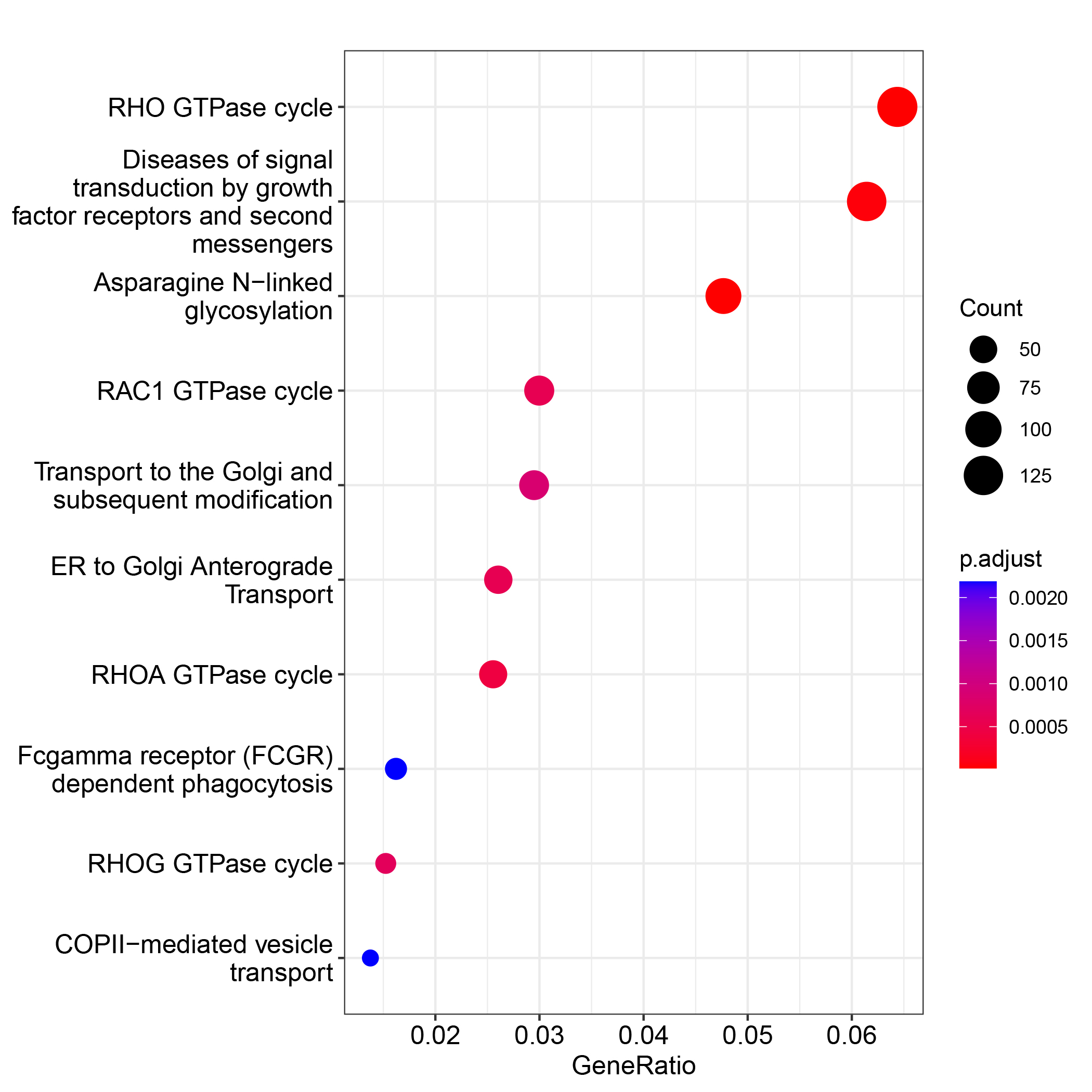


**Supplementary Figure S4A**


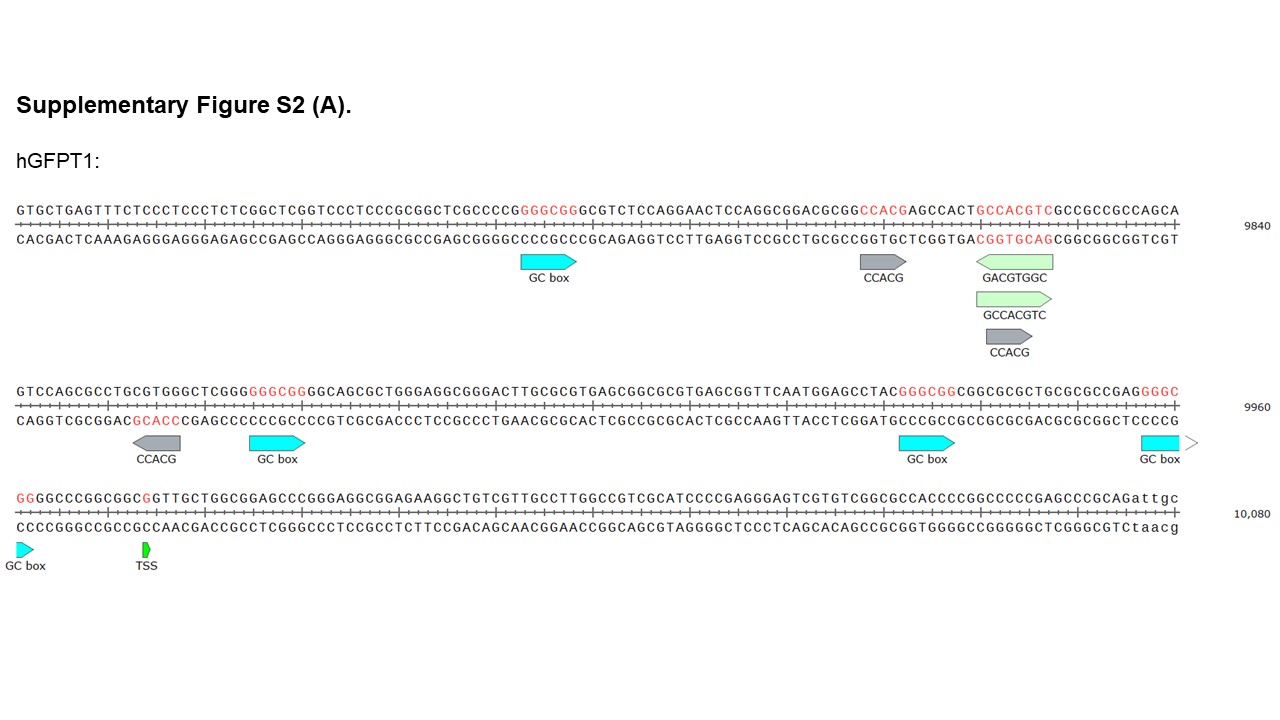


**Supplementary Figure S4B**


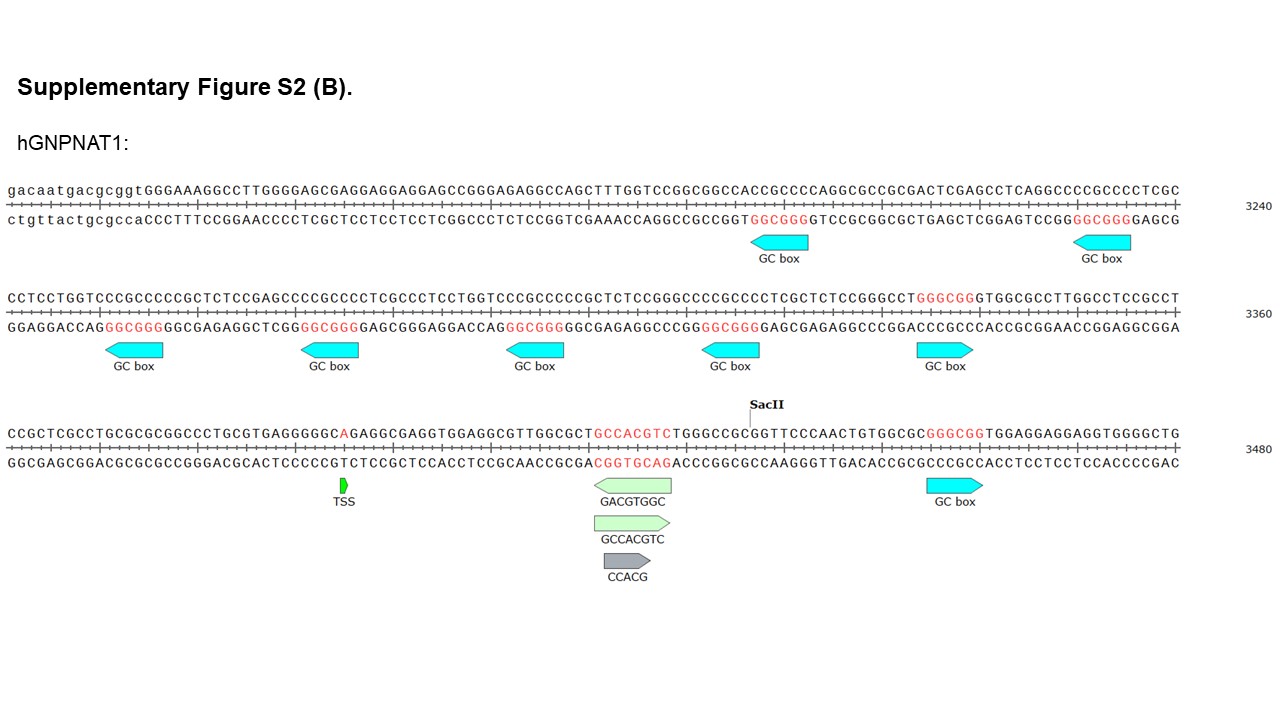


**Supplementary Figure S4C**


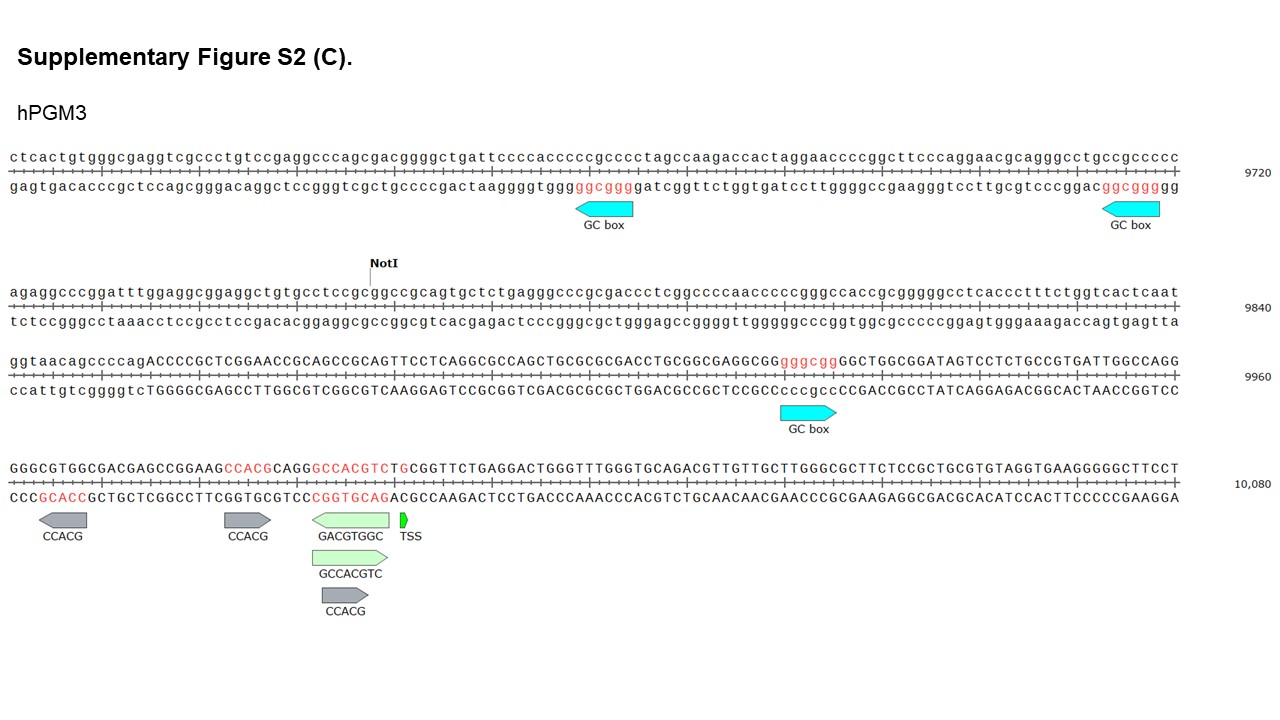


**Supplementary Figure S4D**


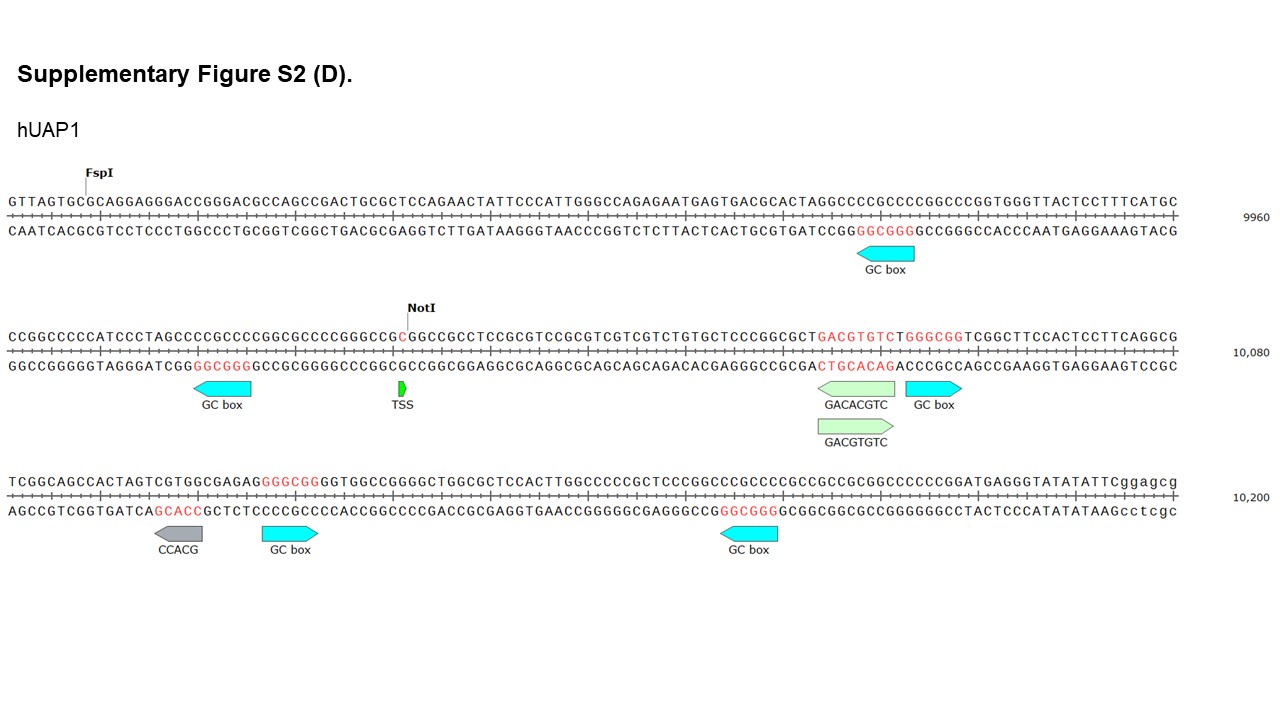


**Supplementary Figure S4E**


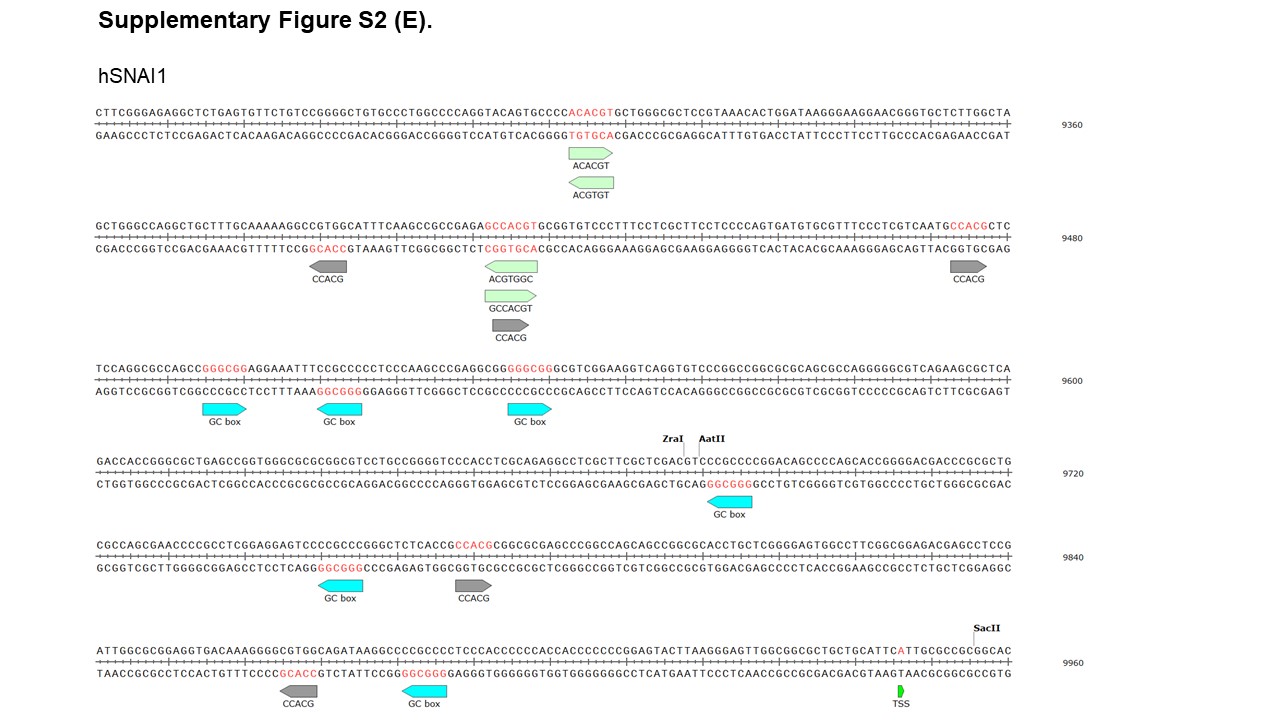

Supplement: gkad077_Supplemental_File [file gkad077_supplemental_file.docx]
